# Stimuli-Responsive Materials for Tissue Engineering and Drug Delivery

**DOI:** 10.3390/ijms21134724

**Published:** 2020-07-02

**Authors:** Sofia Municoy, María I. Álvarez Echazú, Pablo E. Antezana, Juan M. Galdopórpora, Christian Olivetti, Andrea M. Mebert, María L. Foglia, María V. Tuttolomondo, Gisela S. Alvarez, John G. Hardy, Martin F. Desimone

**Affiliations:** 1 Universidad de Buenos Aires, Consejo Nacional de Investigaciones Científicas y Técnicas (CONICET), Instituto de la Química y Metabolismo del Fármaco (IQUIMEFA), Facultad de Farmacia y Bioquímica Junín 956, Piso 3° (1113), Buenos Aires 1113, Argentina; smunicoy@gmail.com (S.M.); inesalvarezechazu@gmail.com (M.I.Á.E.); pablo.e.antezana@gmail.com (P.E.A.); galdo.juan89@gmail.com (J.M.G.); christianolivetti@yahoo.com.ar (C.O.); andreameb@gmail.com (A.M.M.); mlfoglia@ffyb.uba.ar (M.L.F.); mvtuttolomondo@gmail.com (M.V.T.); gialvarez@ffyb.uba.ar (G.S.A.); 2 Department of Chemistry, Faraday Building, Lancaster University, Lancaster, Lancashire LA1 4YB, UK; 3 Materials Science Institute, Faraday Building, Lancaster University, Lancaster, Lancashire LA1 4YB, UK

**Keywords:** stimuli-responsive materials, tissue engineering, drug delivery, biomaterials, thermoresponsive, pH-responsive, light-responsive, redox-responsive

## Abstract

Smart or stimuli-responsive materials are an emerging class of materials used for tissue engineering and drug delivery. A variety of stimuli (including temperature, pH, redox-state, light, and magnet fields) are being investigated for their potential to change a material’s properties, interactions, structure, and/or dimensions. The specificity of stimuli response, and ability to respond to endogenous cues inherently present in living systems provide possibilities to develop novel tissue engineering and drug delivery strategies (for example materials composed of stimuli responsive polymers that self-assemble or undergo phase transitions or morphology transformations). Herein, smart materials as controlled drug release vehicles for tissue engineering are described, highlighting their potential for the delivery of precise quantities of drugs at specific locations and times promoting the controlled repair or remodeling of tissues.

## 1. Introduction

The United States Food and Drug Administration (FDA) defined regenerative medicine as the capacity to facilitate regeneration of parts of the human body, where cells and tissues can be engineered to grow healthy, functional organs to replace diseased ones; new genes can be introduced into the body to combat disease; and adult stem cells can generate replacements for cells that are lost due to injury or disease; tissue engineering and regenerative medicine aim to replace/regenerate tissues from cells and biomaterials [1]. In the case of biomaterials, they can be processed into nanocarriers [2], hydrogels [3,4], and films [5] for drug delivery, wound healing [5,6], tissue engineering, and cell therapy, leading to many emerging and promising regenerative approaches for the treatment of diseases or injuries.

**Figure 1:**
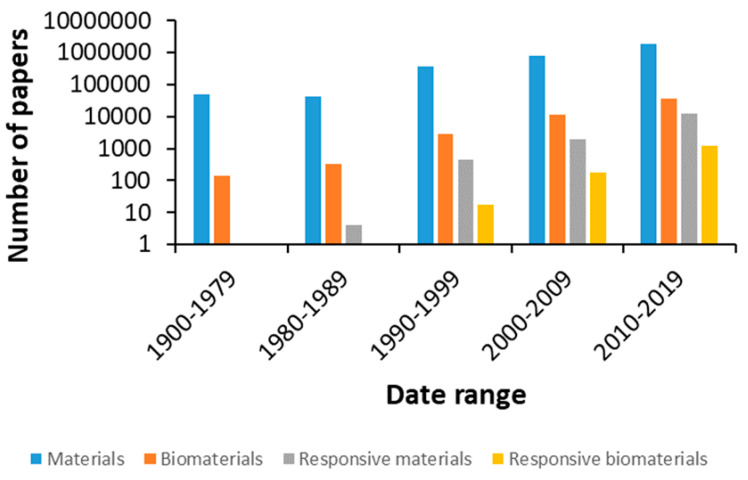
Number of publications on related topics in the Web of Science database with respect to time.

The number of publications in materials science has increased dramatically in recent years, in line with the introduction of new biomaterials and devices to diagnose and treat diseases, aiming to improve our quality of life and contribute to the steady increase in life expectancy (Figure 1). 

Different biomaterials possess different physical and chemical properties and can be processed into a variety of shapes (e.g., films, foams, gels, and particles) [7,8]. They can be used on their own or as part of composites/hybrids in order to impart other functionality and tune their bulk properties, and their surface properties can be tailored through a variety of surface modification techniques [9,10,11]. The ability of a material to respond to different stimuli is related to their physico-chemical characteristics [12,13,14,15,16]. Taking advantage of such features with the recent developments in technology, we expect to be able to control the interaction between the biomaterial and its contents (e.g., cells, drugs) and surrounding environment in response to various stimuli (including but not limited to pH, temperature, redox potential, magnetic fields, and light) [12,13,17,18].

Stimuli-responsive materials have numerous applications in the biomedical field, from drug delivery systems to diagnostics and treatment. The delivery of drugs and genes requires the pharmaceutical compound or gene to reach the site of action at the right time and at an appropriate concentration, traversing obstacles like biological barriers, enzymatic or hydrolytic degradation, and solubility. More often than not, secondary effects arise from non-specific interactions with cells and tissues, so that vehicles that react to specific stimuli would be promising carriers for the targeted delivery of drugs and genes [19,20]. Tissue engineering also faces numerous challenges such as a paucity of renewable sources of functional cells that are immunologically compatible; a lack of suitable materials with the desired chemical composition, mechanical properties, and biological function; and an inability to generate large, vascularized tissues that can easily integrate into the circulatory system of the host with the inherently complexity of native tissues architecture, some of which can be addressed through the utilization of smart responsive biomaterials [21]. In this context, stimuli-responsive nanomaterials have received great attention. Significant progress has been made to tailor nanoparticles with stimuli-responsive properties, which have potential for future therapies for human or veterinary applications. Size, shape, and surface functionalization, as well as modifications, are necessary for active targeting or stimulus-responsive drug release [22].

The stimuli can be internal or external, meaning that they can build up at the site of action or that they could be applied externally to achieve the desired effect. For example, redox conditions and pH vary in the different tissues and between intracellular and extracellular compartments. The properties of redox polymers (ionic, electrical, optical, mechanical, or chemical) change depending on their oxidation state, offering potential for inclusion in actuators, biosensors, and drug delivery systems [23] The review of Guo and coworkers summarized the state-of-the-art of knowledge on reduction/oxidation responsive polymeric drug carriers (specifically focusing on functional groups employed for this end goal) [24]. Drug delivery and tissue engineering strategies based on electroactive materials represents an innovative field of research [25]. The effects of electrical stimulation on cell growth and differentiation and tissue growth has led to interest in using piezoelectric scaffolds for tissue repair [26], influenced by the inherent piezoelectric properties of bone [27], and studies showing enhanced bone regeneration in response to the use of piezoelectric biomaterials [28]. Consequently, piezoelectric materials have begun to find a variety of biomedical applications, including drug delivery and tissue engineering applications [29,30,31]. Of particular interest is the ability of smart polymers to differentiate between the redox potential in tumors and normal tissues (with the former exhibiting 4-fold higher glutathione concentrations), or respond to the presence of reactive oxygen species (ROS), believed to play a role in diseases like cancer, heart injury, and arteriosclerosis [24]. Similarly, pH responsive polymers bearing ionizable acidic/basic residues can be employed in drug/gene delivery, sensors, and membranes [32]. They are of interest as it has been shown that the pH is altered in pathological conditions such as cancer, inflammation, and infection and their ability to respond to changes in the pH by undergoing changes in surface activity, chain conformation, solubility, and configuration has led to the development of several drug delivery systems and wound dressings [33,34,35]. For instance, the variability of pH values between 5.6 and 7.0 in tumor masses has inspired the development of new pH-responsive materials [36]. The pH spectrum observed in different sites within the body in physiological conditions also provides attractive targets for use in biomedicine [37], wherein pH-responsive carriers may be able to target a specific area in the body and release their bioactives with a high therapeutic impact and minimal side-effects [38].

To generate magnetically responsive materials, magnetic nanoparticles can be incorporated into scaffolds for drug delivery, tissue regeneration, and artificial muscles. Through the application of a magnetic field, the nanoparticles are able to transmit a force to the surrounding material and/or cells triggering a response. Mechanical stimulation resulting from the deformation of the magnetic scaffold can lead, for example, to an increase in GAG expression and stimulate differentiation of stem cells to chondrocytes for cartilage repair, promote axonal extension and cell migration to guide neuronal regeneration or induce localized hyperthermia in cancer therapy [39]. In addition, light is an attractive source to trigger a response, as its intensity and wavelength can be controlled to impact specific areas of tissue. Light responsive moieties can then be incorporated into the structure of polymeric materials or molecules bearing light responsive groups can be introduced into a non-light responsive material to induce the release of pharmaceutical compounds or cause shape changes upon exposure to light [40]. Furthermore, thermoresponsive polymers that react to temperature enable the use of polymer networks for the release of drugs at specific temperatures [41]. These materials have been extensively reviewed [42,43], particularly thermoresponsive polymers with interesting lower critical solution temperature (LCST) and upper critical solution temperature (UCST) behaviors in water, the chemical and physicochemical characteristics, and their uses and applications as drug delivery systems, hydrogels, and surfaces for cell growth, among other biomedical applications [42]. Alternatively to the design, mechanism, and behavior of thermoresponsive materials and their combination with other features, namely pH-thermoresponsive or photothermoresponsive materials among others, and their applications in different fields of biomedicine [43], and to complement the existing literature, in this review, we will focus on natural and synthetic thermoresponsive polymers and the results of their application in vitro and in vivo as biomedical materials. 

Interestingly, polymers can be further engineered to tune the response to various stimuli. There are different strategies of molecular design for the incorporation of appropriate responsive building blocks [44]. Moreover, the integration of polymers with different functional groups has allowed the development of multi stimuli-responsive materials [12]. These properties would be further employed to trigger the release of therapeutic molecules in biological environments with different characteristics. Thus, the precise and controlled drug delivery of multi stimuli-responsive carriers may provide new treatment options [45].

Recent articles tend to focus on a particular material, such as nanogels [46], hydrogels [47,48], peptide-based materials [49], and synthetic polymers [50], or examine the major advances on the use of a specific external stimulus including pH, temperature, light, electric field, magnetic field, and ultrasound, in the design of smart materials for medical applications. This review focuses on the materials employed in the design of redox, pH, magnetic field, temperature, and light responsive biomaterials. Recent applications and future perspectives of different stimuli-responsive biomaterials, especially used as delivery systems of bioactive molecules and tissue engineering scaffolds, are covered. This article thereby provides an overview of the latest advances in the development of multifunctional and stimuli-responsive scaffolds for biomedical applications focusing on drug delivery and tissue regeneration.

## 2. pH-Responsive Materials

In diseased, inflamed, and infected tissues, the pH may be decreased due to dysregulated metabolism or irregular angiogenesis, which cause the rapid shortage of oxygen and nutrients, that results in a shift toward glycolytic metabolism [51]; consequently, variations of the pH in different organs, tissues, and intracellular compartments can be considered during the design of nanomaterials with the ability to release a therapeutic agent at a target site in response to pH [52]. 

The pH of extracellular organelles and the bloodstream is typically 7.4, while the pH is 1–3 in the stomach and 6.6–7.5 in the duodenum and ileum of the gastrointestinal tract. The range of pH of the intracellular subendosomal and lysosomal organelles is 5.5–6.8 and 4.5–5.5, respectively [53]. Consequently, there is a broad range of pH values within the human body in physiological or pathological states which can be considered when developing stimuli-responsive delivery systems. Differences in the pH can lead to the modification of crosslinking processes (important for injectable hydrogels and self-healing materials); the protonation or deprotonation of acidic/basic groups can generate distinct interactions between a therapeutic agent and a material causing a defined release profile, potentially in a particular tissue/cell.

**Figure 2:**
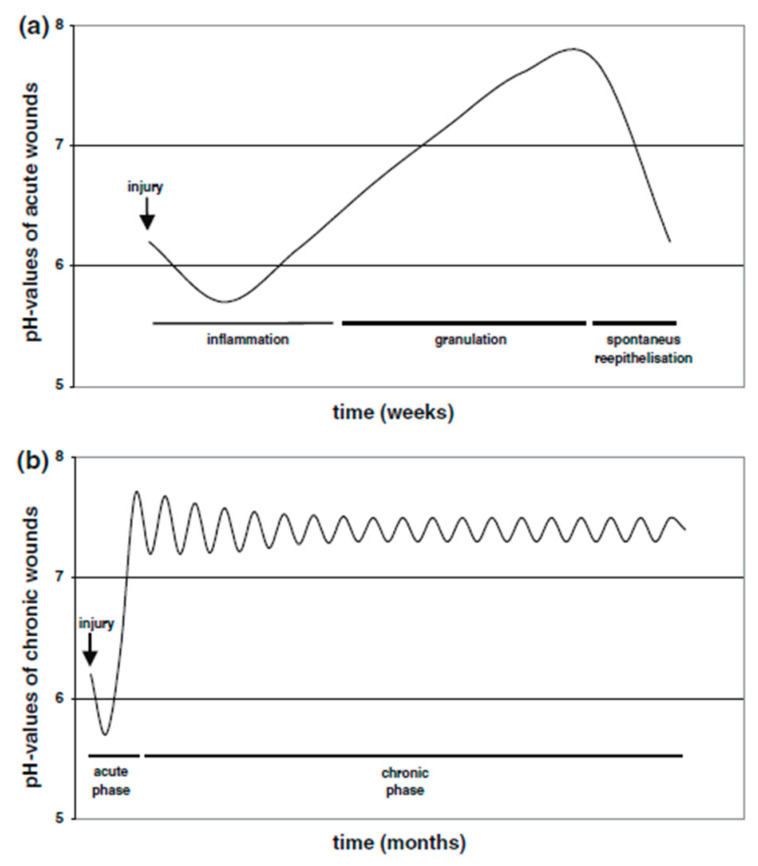
(**a**) Course of pH milieu in acute wounds. (**b**) Course of pH milieu in chronic wounds. Reprinted by permission from Springer Nature, Arch Dermatol Res, in [57], Copyright (2006).

Hydrogels can be engineered so that their physicochemical properties such as their stiffness, degradation, and porosity can directly influence the fates of the encapsulated cells including their migration, proliferation, differentiation, and communication. Furthermore, stimuli-responsive hydrogels could improve the in vivo therapeutic efficacy and also provide new therapeutic pathways [54]. The delivery of therapeutic molecules in nanocarriers to target cells and tissues is also important in tissue regeneration. The delivery of genetic material has the potential to promote the functions of target cells through plasmid DNA and related constructs, or downregulate functions by the reduction/silencing actions. Additionally, a drug in a nanocarrier has the capability to reduce the quantity that is required for achieving therapeutic efficacy, possibly avoiding toxic side effects [55]. Moreover, such carriers can achieve an effective site-specific delivery of drugs or growth factors by exploiting physiological conditions (e.g., pH) in vivo [56], increasing the possibilities of an improved regeneration process or drug therapy. Infected tissues often have an acidic environment, however, it is important to note that the pH of cutaneous wounds is dynamic and displays a good correlation with the stage of the wound healing process (Figure 2), the inflammation stage is acidic, granulation shifts to an alkali pH, and the remodeling phase returns the skin to its initial pH [57]. 

To obtain pH-responsive wound dressings, materials are crosslinked by three polymerization methods: a step growth thiol-ene photoclick reaction, a chain growth by UV polymerization, and mixed mode of the step growth and chain growth mechanisms, to generate materials where the crosslinking density, mechanical and swelling properties could change with the different cross-linking mechanisms. The resultant hydrogels exhibited tunable mechanical properties, swelling ratios, and pH sensitivities without affecting their degradation behavior or their in vitro cytocompatibility with NIH/3T3 fibroblasts, suggesting that these hydrogels may be useful as stage-responsive wound dressings [58]. 

One of the principal challenges in wound healing is the lack of cell recruitment, cell infiltration, and vascularization. Scaffolds which can expand according to the pH may modify oxygen and nutrient transport and cell density, enhancing cell deposition and survival. Consequently, they developed pH-responsive HEMA (2-hydroxyethyl methacrylate)/DMAEMA (dimethylaminoethyl methacrylate) scaffolds via photopolymerization at different molar ratios (10/90, 20/80, or 30/70, mol/mol). They observed that the swell ratio increased with the density of protonated amine groups in DMAEMA. Indeed, the 30/70 scaffold expanded ≈80% in comparison to HEMA at pH 5.5. The swelling behavior of the pH-responsive scaffolds under acidic conditions resulted in an increased oxygen penetration and cell infiltration, which was confirmed by finite element modelling. Furthermore, their results suggested that the 30/70 scaffold could lead to a pro-healing milieu of cytokines and growth factors that could result in significant levels of granulocyte tissue formation and vascularization relative to HEMA [59].

Materials that have ionizable groups are the major candidates for the development of pH sensitive materials, and it is possible to select less conventional strategies to prepare this kind of material. Mesoporous bioglass (MBG) can be used as pH-responsive carriers. The MBG was loaded with metformin hydrochloride (MH) as a model drug and SBF (simulated body fluid) was added at different concentrations and periods of time to cap the pores of the MBG with hydroxyapatite (HAp) and restrict the release of MH. The authors observed that the surface area, pore volume, and pore size decreased after drug loading and HAp mineralization. Moreover, with a longer mineralization time and a higher ion concentration, the pore also decreased, suggesting that a longer mineralization time and a higher ion concentration could benefit the HAp mineralization. Due to the degradation of the HAp of the pH-responsive carrier in acid environments, it was observed that the release profile of MH was modified by the mineralization time and the ion concentration of media while the drug loading efficiency remained constant in every condition. This indicates that this pH responsive material developed is a promising carrier of therapeutic agents involved in the treatment of inflammatory sites and tumors [60].

**Figure 3:**
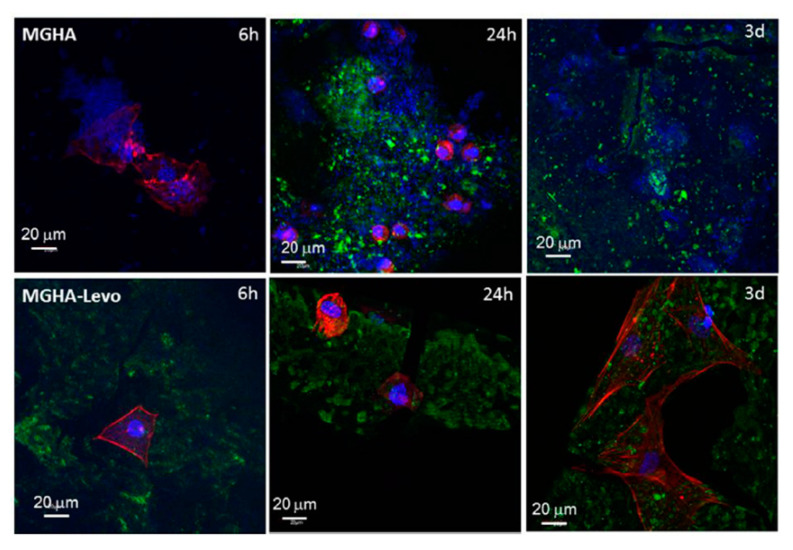
In vitro competitive co-culture MC3T3-E1/*S. aureus* after 6 h, 24 h and 3 days of incubation onto MGHA and MGHA-Levo 3D scaffolds. Material refraction in green, preostoblastic nuclei and bacteria in blue (DAPI) and actin-fibrous of preosteoblast cytoplasm in red (phalloidin). Reprinted from reference [61], Copyright (2018), with permission from Elsevier.

Infections in tissues slow regeneration processes. As mentioned before, the pH in a tissue with an infection is commonly decreased, and multifunctional-therapeutic three-dimensional (3D) scaffolds can be used for the management of bone infection and to support tissue regeneration. They prepared a nanocomposite formed by nanocrystalline apatite embedded into a mesostructured SiO_2_–CaO–P_2_O_5_ glass wall (MGHA) by rapid prototyping. The scaffolds were loaded with levofloxacin, a quinolone that has different protonation states (cationic, zwitterionic, and anionic states) according to the pH, each one with distinct interactions between the levofloxacin (levo) and the silanol groups of the mesoporous matrix. Indeed, these interactions resulted in different in vitro release profiles. Their findings revealed a significantly higher release in an acidic pH environment (pH 5.5 and 6.7) than physiological conditions (pH 7.4), which followed a slow and sustained drug release. To analyze the possibilities of the scaffold for the treatment and prevention of bone infection, an evaluation of the multifunctional capability was performed by in vitro co-culture assays of MC3T3-E1 osteoblast-like cells/*S. aureus* in the MGHA and MGHA-Levo 3D scaffold surfaces. The confocal images obtained after 6 h of culture indicated the absolute absence of bacterial population adhered to the material surface and osteoblasts were attached and growing. The adequate cell colonization of the MGHA-Levo scaffold and the addition of the quinolone in the material which could prevent a possible infection indicate promising features in these potential therapeutic platforms (Figure 3) [61].

**Figure 4:**
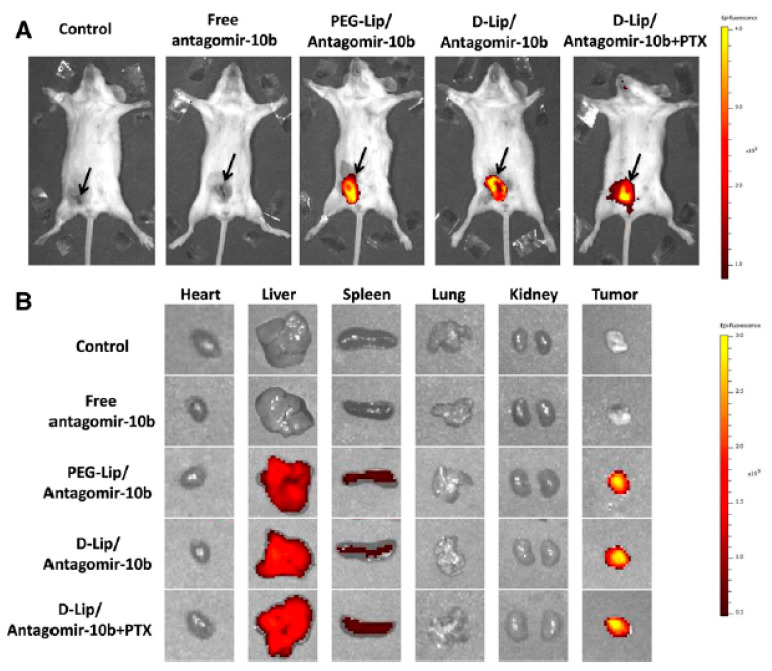
Representative in vivo (**A**) and ex vivo (**B**) images of 4T1 tumor-bearing BALB/C mice 24 h after injection of Cy5 labeled antagomir-10b-loaded liposomes. The black arrows in (**A**) indicated the location of tumors. Reprinted from [65], Copyright (2015), with permission from Elsevier.

The construction of stimuli responsive materials by hydrophilic modification, bioconjugation, or targeting functionalization, with a detailed safety-analysis in small or large animal models could be a method to overcome common barriers like chronic toxicity, long-term stability, the understanding of their biological and physiochemical properties, biodistribution, circulation properties, and targeting efficacy in vivo [62]. In this context, a pH-responsive anti-microbial peptide-mediated liposomal delivery system was developed as a carrier of antagomir-10b and paclitaxel (PTX) for the treatment of murine metastatic mammary tumor models. The antagomirs are a kind of single-stranded RNA analogues that are chemically modified, conjugated with cholesterol to enhance their stability and functioned by hybridizing and repressing the activity of a mature miRNA [63], and the drug paclitaxel is a well-known cytotoxic reagent. They used a pH-dependent antimicrobial peptide named [D]-H6L9 reported previously [64], and tethered it on the surface of liposomes. The histidines in D-H6L9 which have a pKa around 6.5, could be protonated in an acidic tumor environment (which can be as low as 6.0) and a strong membrane lytic effect could thus be activated, leading to the escape of liposomes from the lysosomes and the decrease of antagomir-10b expression. The in vivo and ex vivo fluorescence imaging showed that the [D]-H6L9 modified cationic liposome (D-Lip) could reach 4T1 tumors indicating a good targeting efficacy in vivo of antagomir-10b and PTX. These results imply that D-lip could be a promising vehicle for the management of metastatic tumors [65] (Figure 4).

Hydrogen bonds can be used to tune non-covalent intermolecular interactions in a pH responsive fashion, which is potentially useful for the preparation of self-healable hydrogels for drug delivery and soft tissue regeneration. Self-healing pH-sensitive hydrogels based on cytosine (C) and guanosine (G) modified hyaluronic acid (HA) via hydrogen bonding, with 1,6-hexamethylenediamine (HMDA) as a bridging unit between the nucleobase and HA. The polymer self-aggregation in these types of hydrogels has been attributed to environmental factors such as pH and temperature, transforming them in good variables for the design of new scaffolds or drug delivery systems. Indeed, the authors observed in their microstructure analysis that a higher gelator concentration and a stronger hydrogen bonding between guanosine and cytosine units resulted in the formation of gels with smaller pore diameters. They studied their pH-sensitivity and observed that all three types of hydrogels can be formed only at pH 6–8; logically, the hydrogels with lower concentrations degraded faster than the hydrogels of the higher concentration, however, all the hydrogels had good stability at physiological conditions showing their potential for use in the future as drug carriers or tissue engineering materials [66].

Sometimes, single responsiveness is not able to achieve the desired goals in a physiological or pathological microenvironment. To optimize, multi-stage pH responsiveness materials are emerging. These materials are engineered with different components, which have different sensitivity to pH changes [45], for example, electrospun core–sheath fibers with controlled multi-pH responses within the physiological pH range [67]. This and related research aim to produce new multi-stimuli-responsive materials with active components for drugs or even sensors for targeted disease providing a real-time sensing of various threats. Possibly in the future, these materials will be included in the field of tissue engineering due to their high clinical potential. These materials which are intended to ‘‘sense’’ the surrounding physiological environments and enable on-demand release of encapsulated therapeutic cargos into highly specific targets may optimize the actual therapies in a clinically relevant way. Table 1 summarizes key examples of pH responsive materials.

## 3. Thermoresponsive Materials

Thermoresponsive materials change their physical properties or present conformational changes in response to temperature variations. These materials are used for biomedical applications including drug delivery and tissue engineering among many others [68,69,70,71,72]. However, the transition-state temperature depends on the solvent interaction with the polymer and the hydrophilic/hydrophobic balance. Polymer thermoresponsive properties can be changed by adding reactants to the polymer/solvent system. Some additives are co-polymers, surfactants, co-solvents, plasticizers, and salts. Therefore, additives can alter the solvent quality and therefore can alter the polymer–solvent interactions [73,74,75,76]. 

In this section, we will consider two major groups of materials categorized as natural or synthetic based on their origin. Thermoresponsive materials can also be classified according to their response to temperature changes: polymers that become insoluble above a critical temperature called lower critical solution temperature (LCST) and polymers that become insoluble below a critical temperature called upper critical solution temperature (UCST) [77]. Natural thermoresponsive polymers are, for example, gelatin, agarose, and pectin, whereas synthetic polymers include poly(N-alkyl substituted acrylamides), poly(N-vinyl-alkyl-amides), poly(ethylene glycol)-poly(propylene glycol)-poly(ethylene glycol) copolymer (PEG–PPG–PEG), poly(ethylene glycol)-poly(d,l-lactic acid)-poly(ethylene glycol) copolymer (PEG–PLLA/PDLA–PEG), among many others.

### 3.1. Natural Thermoresponsive Materials

An extensively researched thermoresponsive natural polymer is gelatin. At temperatures below 25 °C, gelatin solutions with sufficiently high concentrations solidify due to the formation of a sample spanning three-dimensional network. When the temperature is raised above approximately 30 °C this rigid three-dimensional network melts. To achieve a good thermoresponsive material for wound healing and tissue engineering, the sol-gel transition temperature must be near body temperature. In several works this temperature (37 °C) is achieved by adding other polymers or plasticizers [77]. Polydiolcitrate-gelatin based scaffolds with thermoresponsive properties are a potential delivery system for bone formation from BMP9-transduced mesenchymal stem cells [78]. PNIPAAm/gelatin nanofibers for thermoresponsive drug release of doxorubicin were developed using 1-ethyl-3-(3-dimethyl-aminopropyl)-1-carbodiimide hydrochloride and N-hydroxysuccinimide as crosslinking agents [79]. Moreover, gelatin nanoparticles were synthesized for doxorubicin drug delivery [80]. 

A promising new thermoresponsive material for tissue engineering and bone reparation are chitosan/β-glycerophosphate based hydrogels. Chitosan is a polymer consisting of glucosamine and N-acetylglucosamine, obtained by deacetylation of chitin present in shells of shrimp and other crustaceans. Chitosan itself is not a thermoresponsive material. Thermoresponsive behavior can be achieved by the addition of β-glycerophosphate. Chitosan/β-glycerophosphate based biomaterials present a LCST near body-temperature. At room temperature the mixture is in liquid state and when this mixture’s temperature reaches body temperature it forms gels. However, this thermoresponsive behavior can be controlled by the addition of copolymers or manipulating the reactant proportions. Thermosensitive chitosan/β-glycerophosphate hydrogels can be used for the sustained delivery of venlafaxine hydrochloride [81]. Thermosensitive chitosan/collagen/β-glycerophosphate hydrogels can be used for soft tissue regeneration: the optimal sol-gel transition temperature could be achieved by adjusting the chitosan/collagen ratio, and the incorporation of β-glycerophosphate increased the biocompatibility towards L929 cells [82]. Thermosensitive injectable hydrogels composed of chitosan/β-glycerophosphate/hydroxyapatite have osteogenic properties, bone induction, bone conductibility, good cellular compatibility, and uniform pore structure to support the adhesion, growth, and osteogenic differentiation of human dental pulp stem cells generating a potentially useful bone tissue engineering material [83].

Cellulose is a linear homopolymer polysaccharide consisting of D-glucopyranose units joined together by β(1⟶4-glycosidic) bonds. Extensive intramolecular and intermolecular hydrogen bonding present in cellulose leads to its insolubility in water. Various cellulose derivatives have been prepared by etherification of the hydroxyl groups of cellulose producing water-soluble derivatives such as methyl cellulose and hydroxypropylmethyl cellulose. Methyl cellulose is a cellulose derivative that has been widely investigated for drug delivery, tissue engineering, and biomedical applications. It has thermoreversible gelation properties in aqueous solutions, with a LCST of 60–80 °C, although, by the addition of copolymers its value can be diminished. HPMC is a partly O-methylated and O-(2- hydroxypropylated) cellulose. The presence of methoxy residues are responsible for gelation but the hydroxypropyl residues have been reported to alter its sol-gel transition temperature. 

Thermoresponsive hydrogels based on methyl cellulose for cell-sheet engineering via 3D-printing can be tuned to display LCSTs near body temperature [84]. Methyl cellulose-based thermoreversible hydrogels (optionally including type-I collagen to improve cell proliferation and growth) can support the adhesion and growth of stem cells in culture [85]. Thermosensitive and mucoadhesive eyedrops containing platelet lysate (a hemoderivative rich in growth factors) for the treatment of corneal lesions by employing hydroxypropylmethyl cellulose associated with chondroitin-6-sulphate sodium, where the addition of chondroitin-6-sulphate sodium resulted in a LCST between 32 and 35 °C [86].

Elastin-like polypeptides (ELP) were designed after elastin and more specifically its precursor tropoelastin. Tropoelastins are water-soluble proteins that are cross-linked to form the insoluble elastin found in the extracellular matrix. Different kinds of topologies of elastin are also responsible for cell adhesion, proliferation, and/or differentiation. These materials have two big advantages: their biocompatibility and their practical synthesis. Conventional peptide synthesis is currently too expensive to produce ELPs in bulk quantities, and the best current route is recombinant production, yielding ELPs that exhibit LCST phase behavior (below their LCST an ELP is soluble in aqueous solution and above this temperature the ELP forms insoluble coils); and the molecular weight and concentration of polypeptides can alter their LCST [87].

### 3.2. Synthetic Thermoresponsive Materials

**Figure 5:**
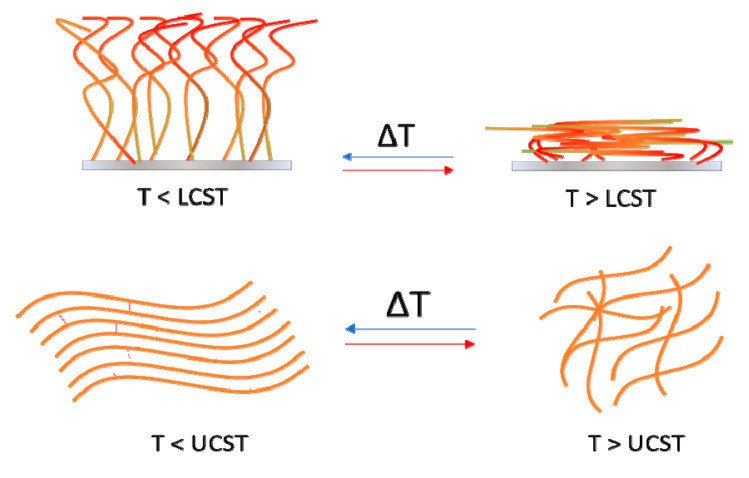
Representative scheme of PNIPAAm gelation at temperatures above its lower critical solution temperature (LCST) (top) and below its upper critical solution temperature (UCST) (bottom).

The most well-known thermoresponsive polymers are based on PNIPAAm (Figure 5), a poly(N-alkyl substituted acrylamide). PNIPAAm presents adjustable water solubility upon heating and cooling processes, having a reversible phase transition from a hydrophilic coil state to a hydrophobic globule state near body temperature (LCST near 30–37 °C). PNIPAAm based polymers are widely used for biomedical purposes such as drug delivery, tissue engineering, and gene therapy (e.g., thermoresponsive polyplex micelles with PEG shells and PNIPAAm layers to protect DNA cores for gene therapy) [88]. Thermoresponsive polymer-coated magnetic nanoparticles loaded with an anti-cancer drug (doxorubicin) using magnetic drug targeting followed by simultaneous hyperthermia and drug release [89]. A bioink for articular cartilage was synthesized by mixing PNIPAAm grafted hyaluronan with methacrylated hyaluronan to develop high-resolution layer-by-layer printed scaffolds with good viability [90]. Encapsulation of human adipose-derived stem cells (ASCs) in hydrogels, further confirms that the AHA-g-PNIPAAm copolymers preserved the viability of the entrapped cells, thus were non cytotoxic. The swelling equilibrium, enzymatic degradation, and cytocompatibility of copolymer hydrogels were dependent upon weight ratios of PNIPAAm. Encapsulated human ASCs possessed spherical morphology and demonstrated that the AHA-g-PNIPAAm copolymer hydrogel allowed cell survival. Furthermore, preliminary in vivo studies in mouse of the AHA-g-PNIPAAm copolymer hydrogel with PNIPAAm (53%) demonstrated biocompatibility. These studies indicate that the thermosensitive AHA-g-PNIPAAm hydrogels have potential application in adipose regeneration, as well as in other soft tissue engineering applications [91].

Hydrogels have shown great potential for tissue engineering applications mainly due to their ability to encapsulate living cells within 3-dimensional, biomimetic microenvironments. Hybrid scaffolding systems that include a thermosensitive hydrogel, poly(ethylene glycol)-poly(N-isopropylacrylamide) (PEGPNIPAAm), and a biodegradable polymer, poly(e-caprolactone) (PCL) were both electrospun into a microfibrous self-supporting hybrid scaffold. The reverse thermosensitivity of PEG-PNIPAAm allowed its hydration and dissolution upon cell seeding within a scaffold of PCL microfibers while maintaining the overall hydrogel shape at room temperature. Cell encapsulation occurs after a subsequent elevation of the temperature to 37 °C, which induced the hydrogel’s phase transition to a gel state. The hybrid material promoted chondrogenic differentiation of human mesenchymal stem cells (hMSCs) based on chondrocytic gene and protein expression, and the resulted superior viscoelastic properties of the constructs. These authors provided a way to create a scaffold that enables a facile, single-step cell seeding process to inoculate cells with the potential for cartilage tissue engineering applications [92]. A hybrid natural-synthetic thermoresponsive material to treat cartilage disfunctions by using chitosan (CS) and PNIPAAm (CS-g-PNIPAAm) was used as a carrier for proliferation and differentiation of mesenchymal stem cells (MSCs). Different microstrip widths were produced to mimic the superficial zone of natural cartilage and evaluate cellular alignment and elongation. After 28 days incubation in chondrogenic medium, encapsulated MSCs within the hydrogel incremented the secretion of glycosaminoglycans (GAGs) and collagen. Histological and immunohistochemical analysis confirmed chondrogenic differentiation. Moreover, cells encapsulated within 50 mm wide microstrips were aligned in a more organized way than those in unpatterned supports. The authors conclude that the cell shape and organization of the microengineered supports resembled the superficial zone of cartilage, while the unpatterned constructs mimicked the middle zone [72]. 

Many researchers have employed nonlinear PEG-based thermoresponsive polymers as an alternative to poly-N-isopropyl acrylamide. The great interest in PEG-based thermosensitive materials is mainly due to the possibility of creating more sophisticated structures, of tuning the transition temperature, and other characteristics such as water-solubility and biocompatibility [72]. Thermosensitive hydrogels based on polyisocyanopeptide (PIC) are interesting because they are liquid below 16 °C and form gels above room temperature. The mechanical properties and architecture of PIC gels are similar to collagen and fibrin, including the characteristic stiffening response at high strains. Their thermoresponsive behavior is reversible and they are biocompatible due to enhanced cell binding capabilities. A recent work compared full thickness dorsal skin wounds in mice treated with PIC gel and PIC-RGD (arginyl-glycyl-aspartic acid peptide, abbreviated to RGD or GRGDS) gel after 3 and 7 days. Equal wound closure rates were found in all groups without foreign body reactions. Moreover, there were no significant differences in myofibroblasts, epithelial migration and macrophages levels, collagen expression, and blood vessels. The authors conclude that these biomimetic PIC hydrogels could be suitable for development into wound dressings [93]. PIC materials would be tailored to meet different requirements of cells by tuning the stiffness or through the grafting of the polymer with short GRGDS peptides using click chemistry. These peptides mimic the binding sites of certain integrins making the hydrogels biocompatible. As a result of the optimization of the PIC polymer properties, they found the optimal concentration of the GRGDS ligand conjugated with the polymer and the ideal stiffness of the hydrogel for efficient cell, tissue, and organ development. Furthermore, it was demonstrated in this work that endothelial cells, fibroblasts, adipose-derived stem cells, and melanoma cells, do survive, thrive, and differentiate in the optimized PIC hydrogels, and these hydrogels. Moreover, the formation of structures like blood capillaries was observed in vitro [94]. 

The development of 3D scaffolds that mimic the biological behavior and organization of the missing organ is important to support the physiological function of tissue in the implanted site. Indeed, the use of 3D bioprinting technology in the field of tissue engineering serves as a powerful tool for building tissue and organ structures. A thermoresponsive water-based biodegradable polyurethane dispersion (PU2) that forms gels near 37 °C avoids the use of the potentially toxic crosslinkers. The stiffness of the hydrogel was tuned adjusting the solid content of the dispersion. Neural stem cells (NSCs) were embedded into the polyurethane dispersions before gelation. The NSCs in PU2 hydrogels had excellent proliferation and differentiation. Furthermore, NSC-laden PU2 hydrogels injected into the zebrafish embryo neural injury model could rescue the function of impaired nervous system, meaning that the function of adult zebrafish with traumatic brain injury was rescued after implantation of the 3D-printed NSC-laden PU2 constructs [95]. Hydrogels based on methacrylated hyaluronic acid (HAMA) and methacrylated poly[N-(2-hydroxypropyl)methacrylamide mono/dilactate] (pHPMAlac)/polyethylene glycol (PEG) triblock copolymers, with optimized bioactivity, mechanical and thermoresponsive properties, embedded chondrocytes and enhanced printability were recently reported to optimize cartilage-like tissue formation. Moreover, mechanical reinforcement was achieved co-printing this with polycaprolactone (PCL). Their results show that HAMA concentrations have a dose-dependent effect on the cartilage matrix production by chondrocytes. HAMA concentrations (0.25%–0.5%) increased cartilage-like matrix production compared to HAMA-free hydrogels, while HAMA concentrations (1%) induced undesirable fibrocartilage production. In parallel, the increase in HAMA concentration correlates well with increase in the material stiffness. These results suggest an optimal hydrogel composition of 19.5% pHPMA-lac-PEG with 0.5% HAMA. This formulation increased cartilage matrix production, limited fibrocartilage formation, and possessed a medium/high Young’s modulus. Moreover, it is adequate for the 3D printing applications. Hydrogel/PCL co-printing enabled the generation of complex 3D constructs with mechanical stiffness in the range of native cartilage. However, the final construct properties are influenced by the coprinting procedure, highlighting the crucial role of the print settings [96]. 

**Figure 6:**
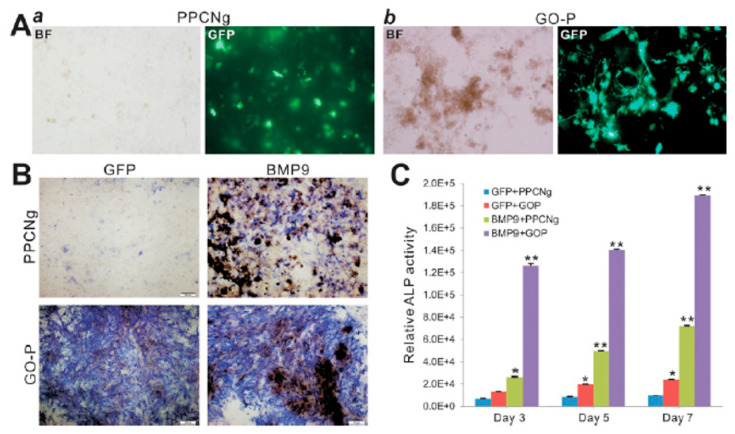
Osteoinductive and osteoconductive activities of the GO-P hybrid scaffold in vitro. (**A**) AdGFP or AdBMP9-infected iMADs were mixed with PPCNg (**a**) or GO-P (**b**) and examined at 48 h after infection under bright field (BF) or GFP fluorescence microscope (GFP). Representative images are shown. (**B**,**C**) ALP activity analysis. AdGFP or AdBMP9-transduced iMADs were mixed with PPCNg or GO-P and seeded in 24-well plates. ALP staining was carried out on day 5 (**B**), while quantitative ALP assay was conducted at 3, 5, and 7 days after infection (**C**). All assays were done in triplicate. * *p* < 0.05 and ** *p* < 0.01 when compared to respective GFP groups. Reproduced from [73]. Copyright © 2018 American Chemical Society.

Bone tissue engineering requires three main constituents: osteogenic factors, osteoprogenitor cells, and osteoinductive/osteoconductive scaffolds. Osteogenic progenitors’ cells are derived from multipotent mesenchymal stem cells (MSCs), which can be obtained from various tissues, including adipose tissue. A citrate-based thermoresponsive hydrogel (PPCNg) and added graphene oxide, obtaining an injectable thermoresponsive hydrogel (GO-P). They demonstrated that cells survive and proliferate in the thermoresponsive hybrid material. In addition, their hybrid material induces alkaline phosphatase activity, BMP9-induced expression of osteogenic regulators and bone markers, and VEGF in MSCs. Moreover, in vivo analysis suggests that BMP9-transduced MSCs entrapped in the GO-P hydrogel form highly vascularized and mineralized trabecular bone (Figure 6). All together, these results highlight the potentialities for bone regeneration of the GO-P hydrogel as a novel injectable scaffold with osteoinductive and osteoconductive properties [73].

Poly(N-vinyl caprolactam) (PVCL) is a biocompatible polymer employed in hydrogels production. Although, PVCL hydrogels are usually non-porous structures with poor mechanical properties. Thus, different cross-linkers are added with consequences in the toxicity. An alternative to improve the mechanical properties is the production of nanocomposites. In this sense, a biocompatible thermoresponsive poly(N-vinyl caprolactam)/clay nanocomposite (PVCL-Clay) hydrogel with improved mechanical properties was developed. Moreover, a macroporous structure was achieved introducing emulsions with N-vinyl caprolactam (VCL) monomer as templates and clay nanosheets as stabilizers. The 3D PVCL nanocomposite was cross-linked by the clay nanosheets. The nanocomposite exhibits improved mechanical properties in comparison to PVCL hydrogels cross-linked by N,N′-methylene diacrylamide. The prepared PVCL-Clay nanocomposite possesses thermoresponsive properties with a phase transition temperature of 35 °C and allows cell culture [97]. 

As highlighted, the applications and materials used to create thermoresponsive biomaterials are extremely wide and this section shows some of the wide spectrum of different techniques available. Table 2 summarizes key examples of thermoresponsive materials.

## 4. Light-Responsive Materials

The strategy of constructing light-responsive smart biomaterials is very attractive to fabricate complex scaffolds for controlling cellular behavior for functional tissue regeneration and stimulating the release of encapsulated compounds [40,98,99,100]. The light stimulus offers some advantages over other stimuli, because it can be imposed instantly and delivered in exact amounts with high precision, providing spatial and temporal control with less invasive techniques [101,102].

Light is an excellent trigger as its intensity and wavelength can be remotely and accurately controlled, quickly switched, and easily focused into specific areas with a resolution of 1 μm. Different wavelengths from UV to NIR have been employed as stimuli. UV light is widely employed in stimuli responsive materials but could be harmful and cannot penetrate deeply into tissues. In contrast, NIR appears to be more suitable for biomedical applications, especially, because NIR is less energetic, causes less damage to biological tissues and can penetrate deeper in the tissues. An interesting review highlights recent biomedical applications of NIR sensitive materials [103].

**Figure 7:**
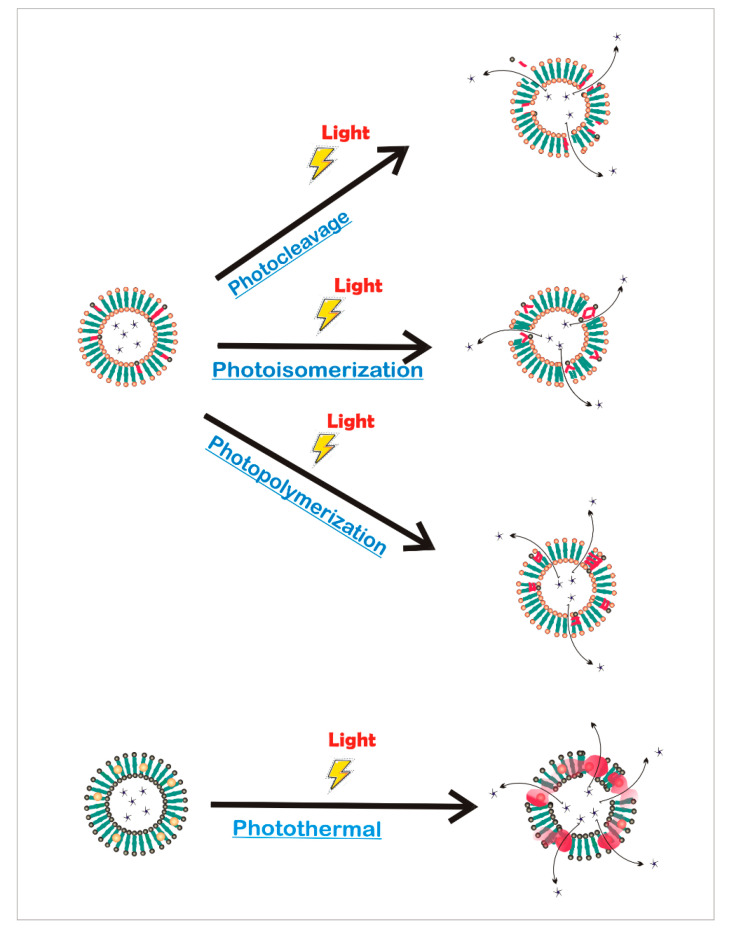
Release from liposomes controlled by photochemical reactions. Photomediated release can be achieved by photocleavage, photoisomerization, photopolymerization, and photothermal reactions.

The spatiotemporal tunability provided by photoreactions has been widely used in different kinds of architecture for both engineer smart therapeutic delivery and creates dynamic cell culture platforms that better mimic living tissues [99]. Light-responsive systems can be classified according to the photochemical reaction involved (Figure 7) [104], photoisomerization, where light induces structural changes; photothermal, where the absorbed photon energy is dissipated via vibrational motion; photocleavage, in which the incident light can break covalent bonds; or photopolymerization, where the crosslinking of a composite occurs in situ with light. 

Photomediated isomerization is the reversible conformational change caused by UV and visible light irradiation, where no chemical bonds are broken. The most commonly used moieties for photoisomer ization reactions are based on azobenzenes [105] and spiropyrans [106]. Photoexcitation of azobenzenes under UV light (365 nm) induces a transition from the *trans* to the *cis* form that has been used to destabilize different drug delivery carriers such as micelles [107], dendrimers [108], and liposomes [109]. The *cis* conformation disrupts the packing of these assemblies by a steric effect and increase in polarity, a long *cis* lifetime is desired to extend the effect of the light stimulation favoring the release of the encapsulated drugs. It has been shown that when an azobenzene derivative is inserted into liposomes, the *trans−cis* isomerization of the azobenzene group can induce defects in bilayers that lead to the release of the entrapped payload [110,111,112]. Azobenzene-glycolipids that were embedded into HSPC/DSPG/Chol liposomes to control temporarily and quantitatively drug release by light stimuli [112], were shown to keep the entrapped drug stable in the dark but release nearly 100% of cargos instantaneously with UV irradiation. It is also possible to make fluidphase photosensitive nonphospholipid liposomes formed by decyl-azobenzyl-triethylammonium and cholesterol sulfate, as a promising approach to control multidose release by photocycling between the *cis* and *trans* azobenzene isomers [113]. This distinct composition confers to these photosensitive nonphospholipid vesicles some advantages over conventional phospholipid-based liposomes, like enhanced impermeability and more chemical stability.

Apart from liposomes, micelles containing photoisomerizable moieties have also been developed [114,115]. For example, photoresponsive micelles of polyglycerol incorporating spiropyran [115] self-assemble into light-responsive micelles where the hydrophobic spiropyran isomerizes to hydrophilic merocyanine upon exposure to UV irradiation, leading to the disassembly of the micelle structures and allowing the release of the hydrophobic content. Besides, this interconversion between the closed spiropyran form and the open merocyanine was successfully used in the building up of a new concept of drug delivery based on smart nematic liquid crystal microspheres [116]. Spiropyran molecules bound to a specific therapeutic drug photoisomerize to merocyanine and the combination of merocyanine-drug molecules can translocate across the liquid crystal barrier. The unique properties of liquid crystalline materials give spiropyran doped liquid crystal vehicles many advanced optical characteristics that provide a new approach to sophisticated delivery of active therapeutics in a time-specific and stimuli-specific manner. 

The reversible photoisomerization behavior of the aforementioned moieties has also been applied for engineering advanced smart biointerfaces for functional tissue regeneration. Polymers and hydrogels have been widely used as biomaterials for the fabrication of medical devices and tissue engineering scaffolds [117,118], as they provide biocompatibility, biodegradability, mechanical properties, hydrophobicity, and crystallinity that are crucial to allow tissue growth. An interesting material was developed by coating strawberry-like silica Janus particles with spiropyran-containing polymer brushes for controlling cell capture and release by UV and visible-light irradiation [119]. This hydrophobic coating showed high efficiency to cell capture due to the specific interaction between the hydrophobic ring-closed of spiropyran form and cell surface fibronectin protein [120]. After UV light irradiation, nearly 94% of captured cells were released from the coatings because of the weak interaction merocyanine-cell fibronectin protein. In this way, the strategy of constructing light-responsive composite coatings provides an inspiration for the design of smart biomaterials for regenerative medicine. In a related manner, azobenzene-containing photoresponsive materials have been used as cell culture supports, opening up new possibilities in the study of the processes involved in the dynamic cell–material interaction and in cell-cell signaling [121,122,123]. Polyacrylamide hydrogels that incorporate an azobenzene molecule for the photoswitchable manipulation of primary human mesenchymal stem cells have been extensively studied for their potential application in tissue engineering and regenerative medicine [124]. They demonstrated the potential of this material as a minimally invasive method to study mechanotransduction and modulate the behavior of mesenchymal stem cells through the alteration of substrate mechanics in response to light stimulation.

Photothermal reactions have also been extensively used to control drug delivery and stimulate tissue reconstruction through the interaction between a plasmonic material and light irradiation [125]. Typically, when conductive materials absorb incident photons, this energy becomes local heating which in turn stimulates drug release. This method has been demonstrated for various light-absorbing moieties, such as molecular dyes [126,127], metallic particles [128], and plasmon resonant gold nanoparticles [129,130,131]. The most commonly reported hybrid platforms for photothermal effect induced controllable drug delivery include colloidal gold encapsulated in liposomes [132]. Gold nanoparticles are popular because they are inert, nontoxic, and have tailorable optical and photothermal properties depending on their size, shape, and surface chemistry [130]. They strongly absorb photons in the Near-Infrared Radiation (NIR) region of the spectrum that are turned into heat by the plasmonic effect of the nanoparticles: the absorbed photons are transformed into phonons, followed by a phonon–phonon relaxation, resulting in an increase of the temperature of the system and by conduction to its surroundings, producing local heat [133].

Taking advantage of these photothermal properties of gold nanoparticles, Lajunen and coworkers formulated biocompatible liposomes with star or rod shaped gold nanoparticles that showed a controlled content release in the cell cytosol after exposure to visible and NIR light [134]. Compared to other studies on light activated liposomes based on the UV light, the lipid formulations they used not only represent an improvement in safety but also enable more efficient light induced drug release. This technology is an attractive option for the treatment of pathological conditions that benefit from specific control of location and timing of the drug release. Another approach to light-activated drug delivery by photothermal heating triggered by near-infrared light was based on the co-delivering of low-temperature-sensitive liposomes with multibranched gold nanoantennas (MGNs) [129]. Co-delivering liposomes and nanoantennas has several advantages over encapsulating the gold nanoantennas within the vesicles: it allows loading a higher amount and a broader range of drugs into the liposomes; the size of the liposomes can be controlled; and co-delivery also enables more nanostructures to be localized in a specific place for enhanced photothermal transduction and drug delivery from liposomes. The synergistic therapeutic effect of mild hyperthermia (≈42 °C) of MGNs with drug delivery from the liposomes here presented will facilitate targeted treatment of multiple diseases by providing a controlled release and minimizing off-target toxicity.

Gold nanoparticles synthesized in a collagen protein hydrogel through a biomineralization-triggered self-assembly yielded light-stimulated protein-based delivery vehicles [135]. The incorporation of gold nanoparticles was able to bridge the collagen fibers, avoiding the use of toxic cross-linkers and thus enhanced the hydrogel strength and stability against higher denaturation temperature. This functional collagen-based hydrogel resulted advantageous as an in vivo injectable material for biomedical applications because it has good biocompatibility and biodegradability, their mechanical properties make it an ideal injectable hydrogel for tissue engineering, and the photothermal responsive behavior is promising for alternative light-actuated drug delivery platforms. 

The photothermal stimulation strategy has also been applied to control cellular mechanisms for the creation of new platforms with possible applications in tissue engineering. As an example, Martino and coworkers studied the photo-modulation of Human Embryonic Kidney 293 cells grown onto conjugated poly(3-hexylthiophene) thin film [136]. They demonstrated that NIR light absorption by the semiconducting polymer leads to the generation of local heating of the cellular environment that affects the electrical properties and conductance of the ion channels present in the cell membrane. This photoexcitation of living cells mediated by polymer absorption appears as a promising tool that can be developed into a platform for cell light-control with many biomedical applications.

Photocleavage is another common approach that has been widely employed in light-induced drug delivery [137]. Photoreactions that involve photocleavage occur when a covalent bond is broken by light irradiation to facilitate the release of the encapsulate molecules [138]. In one case, drug molecules directly attached to a carrier by photocleavable linkers are released when the linkers are cleaved upon light irradiation. Otherwise, photoresponsive moieties are incorporated in the drug carrier and, after the light stimuli, the groups are cleaved, and the drug carrier is broken, causing the therapeutic content to be released. These systems usually use UV and high-energy visible light that have sufficient energy per photon to break covalent bonds. However, these short wavelengths suffer a serious drawback for in vivo application because they cannot penetrate deeply into most tissues due to light scattering and absorbance by intrinsic biological chromophores. Consequently, various strategies have been recently employed to replace UV and visible light with NIR light, which achieves greater tissue penetration [139,140]. 

The most commonly used photocleavable molecule is the ortho-nitrobenzyl (o-nitrobenzyl) moiety [141], for example, micelles based on poly(S-(o-nitrobenzyl)-L-cysteine)-b-poly(ethylene glycol) block copolymers to drug delivery [142]. After irradiation at about 310 and 350 nm, the o-nitrobenzyl groups were gradually photocleaved and the self-assembled micelles became smaller until they completely cleaved, allowing the encapsulated drug released in a controlled manner by changing the light irradiation time. This work provides an attractive strategy not only for the development of photoresponsive polypeptide-based block copolymers but also for the fabrication of photo-stimulated nanomedicine therapies.

The nitrobenzyl group has also been incorporated into liposomes for drug delivery [143,144,145]: the hydrolysis of o-nitrobenzyl upon light irradiation results in the separation of hydrophilic and hydrophobic groups from the amphiphilic phospholipid, causing membrane destabilization and consequent drug release. Liposomes with photocleavable properties could be formed in situ through the grafting of an o-nitrobenzyl-containing azide tail precursor and an alkyne-functionalized lysolipid through the copper-catalyzed azide–alkyne cycloaddition reaction [143]. Photolysis of the included o-nitrobenzyl group changed the molecular structure of the photolabile phospholipids, inducing an increase of the phase transition and permeability of the lipid membrane, and subsequently provoking disruption of the liposome structure and cargos release. This in situ liposome production method combines the design of the precursor and click reaction to prepare photo-responsive liposomes for drug delivery.

As light offers precise real-time spatial and temporal control, there is considerable interest in photodegradable hydrogels as 3D carriers for cells and delivery of therapeutic molecules for applications ranging from wound healing and tissue regeneration to disease treatment [146,147,148]. As a potential alternative to the previously reported nitrobenzyl-based systems, coumarin-based photodegradable hydrogel platforms have been developed [149,150,151]. Coumarin derivatives provide favorable advantages over nitrobenzyl-based linkers, such as high absorption efficiencies, fast cleavage rates, red shift in its degradation wavelengths and affinity for multiphoton-induced reactions [152]. Coumarin moieties can be used to manipulate poly(ethylene glycol) (PEG)-based hydrogel materials at 365 and 405 nm (like nitrobenzyl groups) but also at longer wavelengths between 720 and 860 nm, producing biologically benign byproducts upon photocleavage [149]. Moreover, a short photocage bromohydroxycoumarin crosslinker was used to create light-degradable hydrogels and nanogels [150]. They have shown that the coumarin-based crosslinker breaks by intramolecular cyclization in response to UV and NIR light, enabling rapid degradation of polyacrylamide gels and releasing of small payloads such as iron oxide nanoparticles, a model protein (BSA) and murine mesenchymal stem cells. All of this new versatile chemistry of hydrogels is promising for future tissue engineering studies focused on spatiotemporal patterning of cellular microenvironments, as well as for transport of bioactive molecules in living systems and cell delivery implants for regenerative medicine. 

**Figure 8:**
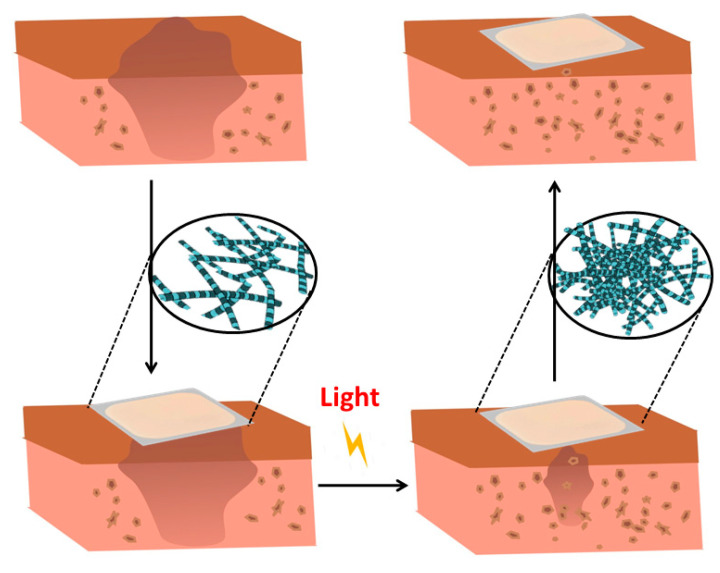
Scheme showing a photoreactive composite for tissue regeneration. Photopolymerization allows to initiate and propagate a polymerization of networks in situ, creating covalently crosslinked hydrogels for tissue reconstruction.

Fabrication of hydrogels and polymers in situ is also attractive for tissue engineering because this allows to form complex shapes that adhere and conform to tissue structures [153]. One advantageous strategy that enables in situ formation of crosslinked polymers and hydrogels is the photopolymerization [117,118]. Photopolymerization allows the initiation and propagation of polymerization of networks in situ, creating covalently crosslinked hydrogels, through the exposure of a photosensitive system composed of unsaturated prepolymers, photoinitiators, and other compounds such as cells and therapeutic molecules, to UV or visible light (Figure 8). The photopolymerization technique has several advantages over conventional polymerization techniques. Firstly, photopolymerization provides tailored temporal and spatial control over the gelation process, occurs at fast curing rates at room or physiological temperature and is a non-invasive method. Moreover, it can be achieved within minutes employing cytocompatible photoinitiators under biocompatible reaction conditions [154,155]. This rapid process provides facile control over the spatiotemporal formation of the gel at relevant length scales, which is important not only to induce the hydrogel crosslinking, but also to introduce specific biochemical functionalities in 3D environments [156]. 

Photopolymerization has been applied to develop a biosynthetic soft tissue replacement composed of PEG and hyaluronic acid (HA) that can be injected and photocrosslinked in situ with transdermal light exposure [157], thereby enabling soft tissue reconstruction by implantation into the damaged site, manipulation into a desired shape, and then crosslinking in situ with light. For this, the authors designed an array of light-emitting diodes that could penetrate up to 4 mm of human skin without any painful side effects. The implanted photocrosslinked PEG-HA in rats presented a dose-dependent relationship with PEG concentration and were able to maintain near their original volume for up to 491 days. The polymer was also tested in humans to confirm the feasibility of the transdermal photocrosslinking approach for implantation in soft tissues of the abdomen. Although an inflammatory response was observed surrounding the materials, this new photocrosslinkable biosynthetic polymer and transdermal crosslinking method is an interesting paradigm for soft tissue contouring and reconstruction.

Some other photocrosslinked hydrogels apart from being suitable for tissue regeneration, are also promising for drug delivery, for example, photocrosslinkable hyperbranched polyesters (HPE) hydrogels with sustained drug release characteristics for cellular therapies [158]. The encapsulation of dexamethasone acetate into the HPE and functionalization with acrylate moieties resulted in the formation of hydrogels with a highly porous interconnected structure and mechanically tough network. The feasibility of using these HPE networks for cellular therapies was then investigated by evaluating drug release, cell adhesion, spreading, and proliferation on hydrogel surface. They demonstrated that HPE hydrogels had a sustained release of entrapped dexamethasone acetate over a period of eight days and the highest crosslinked HPE hydrogels had higher cell adhesion, spreading, and proliferation compared to soft and compliant HPE hydrogels. They further showed that cells readily adhered and proliferated on hydrogel sheets to form a uniform cell layer that covered the entire hydrogel surface, demonstrating their potential use for cellular therapies.

Many promising advances in the development of light-responsive biomaterials have been highlighted. The use of externally manipulated light offers excellent spatial and temporal control for drug delivery and engineering 3D microenvironments for tissue regeneration. In photo-controlled technologies, a variety of light-induced reactions, such as photopolymerization, photoisomerization, and photodegradation, are employed to promote the release of encapsulated drugs and synthesize or introduce changes in hydrogel networks. These technologies are very attractive to manipulate biomaterials features in real time providing further control over cell functions, tissue restoration, and delivery of therapeutics. Table 3 summarizes key examples of light responsive materials.

## 5. Redox-Responsive Materials and Electroactive Polymers

Redox-responsive polymeric materials can respond to biological stimuli generated by the presence of oxidants or reductants in the media and changes in the redox conditions or by the application of an external voltage. The chemical groups which are involved in their redox responsive ability include disulfide bonds, organometallic compounds, viologens, or tetrathiafulvalene. Their biomedical applications in the design of artificial muscles and self-healing materials as well as their delivery properties, mainly for the treatment of cancer, will be discussed in the following section. The application of electroactive polymers with tissue regeneration purposes will also be discussed.

### 5.1. Polymers Containing Disulfide Bonds

This group of polymers is mainly utilized to controlled drug delivery in tumor cells making it possible to control the destiny of the drug which remains inactive in systemic circulation and releases in response to a redox stimulus at the desired sites. For the stimuli responsive controlled delivery, it is considered that the intracellular concentration of reduced substances, mainly glutathione, is approximately 10 mM, but it is only 1−10 μM in fluids outside cells, such as plasma. Glutathione in the cytoplasm takes part in the oxidation of the thiol groups of proteins. It is a tripeptide with a central cysteine which can be in a reduced (GSH) or oxidized form where it forms a dimer with a cysteine from another glutathione molecule (denoted GSSG) [159]. In addition, the intracellular glutathione concentration is altered in cancer cells compared to normal tissues [160,161]. 

For this reason, the synthesis of polymers carrying disulfide bonds is utilized as a strategy for the delivery of antitumoral drugs. For example, a glycolipid-like copolymer based on chitosan oligosaccharide and stearic acid was obtained where the authors incorporated the drug doxorubicin (DOX) attached to the copolymer via a disulfide linkage. These micelles were able to respond to the reductive intracellular microenvironment of tumor cells releasing the drug due to cleavage of disulfide linkers [162]. Other studies performed with the same chemotherapeutic agent used a biocompatible and biodegradable redox sensitive polymersome nanosystem which was based on a polyethylene glycol modified polycaprolactone copolymer with a disulfide linkage in between [163]. Polymersomes are formed through the self-assembling of amphiphilic polymers in aqueous media. They possess a hydrophobic membrane with an aqueous center. This structure allows the incorporation and transport of both hydrophilic and hydrophobic therapeutic agents. Moreover, the membrane can be grafted with different ligands for targeted drug delivery and to introduce stimuli responsive properties. In this way, it is possible to develop polymerosomes that respond to chemical, physical, and biological stimuli [164].

In some materials, it is not necessary to introduce chemical modifications that provide the disulfide bridges, but because of the very nature of the polymers, they can be present in the structure and available for the redox stimuli. This is the case of keratin which contains abundant polar side chains made of disulfide, carboxyl, and amino groups. Keratin nanoparticles exhibited a pH/reduction dual-responsive characteristic as well as charge reversibility under a tumor microenvironment of low pH and high GSH levels [165]. Similarly, keratin grafted poly(N-(2-hydroxypropyl)methacrylamide) copolymer micelles were synthesized for the delivery of DOX making use of the thiol groups of keratin and the amphiphilicity of the graft copolymers to achieve a controlled release of the drug sensitive to GSH levels [166].

While the literature on the use of disulphide bond-carrying compounds in the delivery of drugs for cancer treatment is vast [167,168], less information is available about their application for regenerative medicine.

### 5.2. Ferrocene Containing Polymers

Other polymers contain ferrocene as the key component of their redox responsiveness. These are a group of metal-containing polymers with high chemical stability which in principle can be obtained in two ways: incorporating covalently bound ferrocene into the polymer in the main chain (backbone) or by attachment of ferrocenyl units to pendant groups adjacent to the polymer backbone. The main aspects of their synthesis and structure can be found in recent reviews for both types of polymers [169,170]. As we mentioned before, herein we will focus on their application as redox-sensitive biomaterials or nanosystems intended for drug delivery.

Self-healing materials have gained increased importance in the development of biomaterials. The self-healing process is possible thanks to the introduction of reversible covalent bonds or non-covalent interactions such as hydrogen bonds, hydrophobic, ionic, or host–guest interactions [171,172]. Hydrogels incorporating covalent bonds tend to suffer less degradation and longer function times than hydrogels obtained with non-covalent interactions. The host–guest interaction has been described for the obtaining of self-healing materials which have the unique property of self-repair after physical, chemical, or mechanical damage to recover their original properties. Poly(acrylic acid) modified with cyclodextrins (pAA-CDs) was used as a host polymer and pAA with ferrocene (pAA-Fc) was used as a guest polymer with the aim to obtain this kind of material. The authors found that adding NaClO to the hydrogel decreased its viscosity while continuous addition of GSH to the sol recovered the elasticity, reverting it back to the hydrogel state due to the high affinity of β-CD for the reduced state of the Fc and the low affinity express for the oxidized state of the Fc group (Fc+). In the same way, the electrochemical oxidation of the pAA-6βCD/pAA-Fc hydrogel decreased the elasticity. They also observed the self-healing properties of the materials due to the Fc and β-CD moieties, which form an inclusion complex on the cut surfaces of hydrogels [173]. 

Other materials which do not contain ferrocene in their composition have also been explored as self-healing polymers. For example, hydrogels composed of poly (1-vinyl-2-pyrrolidinone) modified with O-carboxymethyl chitosan and acrylamide, which would have potential applications in regenerative medicine and wound dressing as they observed that two gel pieces of this material could be merged into an integrated one through hydrogen bonding between amides, hydroxyls, and carboxylic groups. Moreover, they focused on accelerating the healing rate of the hydrogels based on the introduction of electrospun cross-linked nanofiber networks containing redox initiators as the healing layer and observed that this layer initiated cross-linking reactions which further form more hydrogel networks [174].

In the field of drug delivery, ferrocene complexes with cyclodextrins have been studied. The hydrophobic cavity of cyclodextrins (CD) is known to form relatively stable inclusion complexes with hydrophobic and size compatible molecules like ferrocene. This methodology has been used by some authors to develop nanocapsules of ferrocene/β-CD-grafted polymers demonstrating that their permeability could be regulated by applying an electrochemical stimulus that changed the stability of the host–guest pairs, thus changing the capsule wall structure [175]. 

### 5.3. Viologens

Polymers which can be modified in a reversible way by oxidation/reduction reactions of the material can be used for the design of artificial muscles. In this case, the change in the redox state of the polymer components consequently produces a reversible change in its volume. The reversible volume change is similar to the one that takes place in the natural muscles during the contraction/ relaxation process and can be translated in controllable movements. 

**Figure 9:**
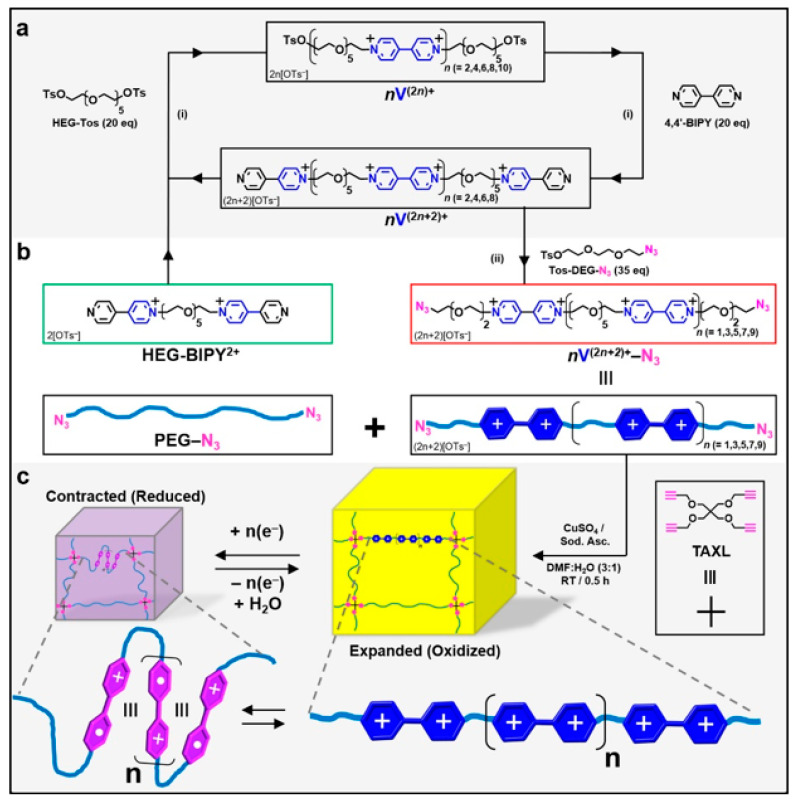
(**a**) An iterative synthesis was used to prepare each even-numbered (*n* = 2, 4, 6, 8, and 10) oligoviologen by (i) alternating between the excessive addition (20 equiv) of tosyl end-capped hexaethylene glycol (HEG-Tos) and 4,4′-bipyridine (BIPY) in MeCN at 130 °C for 12–16 h in a closed reaction vessel. (**b**) The synthetic cycle begins (green box) with a BIPY end-capped HEG (HEG-BIPY2^+^), and the oligomer is grown iteratively, with only intermittent precipitations in MeCN:PhMe, followed by centrifugation in order to isolate each product. At any point in the cycle, the BIPY end-capped precursor can be removed and (ii) functionalized with terminal azide groups (red box) through the excessive addition (35 equiv, MeCN, 130 °C, 20 h) of a tosylated diethylene glycol possessing one azide at its terminus (Tos-DEG-N_3_). (**c**) Synthesis of the click-based hydrogel involves 2 equiv of bis-azide-terminated linkers, where 95 mol % of the 2 equiv is composed of polyethylene glycol (PEG-N_3_) and 5 mol % consists of the oligoviologen (nV(2n) +-N_3_), to 1 equiv of the tetra-alkyne cross-linker (TAXL). Published in [176]. Copyright© 2017 American Chemical Society.

Viologens are dialkyl-4,4′- and 2,2′-bipyridiniums compounds which have an electroactive behavior and they have been used in the synthesis of artificial muscles. In their work, the driving force for network reorganization was the reduced electrostatic repulsion and exclusion of water and counteranions from the material after chemical reduction of the viologen subunits and formation of radical cations. They found that the degree and rate of actuation was markedly improved as more viologen subunits were present and that the process was reversible with a reduction of 9% of the original volume after a few hours [176]. The synthesis of such materials is shown in Figure 9.

As it has been previously described for ferrocene displaying materials, the presence of viologen-cyclodextrin complexes is a very useful strategy for host–guest interactions. Inclusion complexes of β-CD and methyl viologen (MV) as a stimuli-responsive supramolecular bond for the cross-linking of acrylamide gel networks has been studied. They confirmed that the association and dissociation between CD and MV in the polymer chains occurred in response to a redox reaction and that affected the macroscopic properties of the gel, such as viscosity. Furthermore, they compared the properties of single-network, where both CD and MV were present in the same polymeric network, or double-network gels, where CD and MV were included and associated in different polymeric networks [177]. 

### 5.4. Tetrathiafulvalene

Tetrathiafulvalene (TTF) and its derivatives have been investigated as conducting materials which are excellent *π*-electron donors used in the design of stimuli responsive materials. TTFs can be oxidized reversibly under their exposure to an appropriate oxidant and/or reducing agent. Three oxidization states are present in TTFs, the neutral TTF (TTF^0^), which exists under ambient conditions, the cation-radical (TTF^+•^), and the dication (TTF^2+^) state that are prepared by chemical or electrochemical oxidation of the neutral compounds [178].

TTFs are commonly used as molecular redox switches with the aim to generate and control molecular motion. A recent review about artificial molecular machines, which are intended to convert energy into directional mechanical motion on the nanoscale, describes the use of TTFs [179]. TTF has also been used in the development of drug delivery vehicles. The synthesis of a series of PAMAM dendrimers with TTF groups covalently modified at the periphery was studied. Upon redox stimulus, the terminal TTF groups transform to the oxidized form TTF^+•^, which can further interact with cucurbiturils, macrocyclic molecules that behave like molecular capsules, forming inclusion complexes at the periphery of dendrimers. The formation of these complexes after chemical oxidation loosens the structure of the nanospheres and initiates the release of cargo [180]. Amphiphilic polymers featuring a redox active TTF hydrophobic unit and a temperature-sensitive hydrophilic poly(NIPAM) shell which self-associated to form micelles. These micelles were therefore sensitive to both temperature and chemical oxidation of the TTF moiety to a more hydrophilic dicationic state [181].

### 5.5. Electroactive Polymers for Tissue Regeneration

Electroactive hydrogels are mostly used in electroactive tissues like nerves, cardiac tissue, or skeletal muscles. They respond to changes in the electrical field to which they are exposed and are expected to support the contraction-relaxation episodes of these kinds of tissues which could cause hydrogel disruption. They are promising materials in the biomedical field to regenerate damaged tissues as the employment of electrical signals, which are the main physical stimuli present in the human body, can modulate cell proliferation and differentiation [25,182] and potentially also deliver drugs in a controlled way [183,184]. 

Conductive materials have been used mainly as a support for cells and their application in tissue engineering has been extensively studied [185,186,187]. However, the objective of this section is to describe their application in stimuli-responsive biomaterials and not only their use in a passive way as scaffolds. They are electrically conductive with chemical structures characterized by alternating single and double bonds with overlapping pi-bonds which allow the free movement of electrons to give them a conductive behavior. The electronic jumps between chains are facilitated thanks to the presence of dopant agents. 

Polypyrrole (PPy) is a popular choice of conductive polymer for biomedical purposes as it shows excellent biocompatibility and can be electrochemically modulated. For example, it has been investigated as an in vitro system to study the presentation of cytokines to hematopoietic cells. In this work, interleukin IL-3, known to affect hematopoiesis, has been immobilized on polypyrrole films to study its effect on a bone marrow-derived progenitor cell line and a difference in cell viability was observed between the oxidized (0.2 V, oxidation) or reduced (0.7 V, reduction) states. It was believed that the conformational changes between a collapsed or fully extended protein structure altered the receptor/IL-3 interaction and were responsible for the differences in cell viability after application of an external voltage [188]. 

Apart from PPy, the other two most investigated conductive polymers are polyaniline (PANI) and poly (3,4-ethylenedioxythiophene) (PEDOT) [189]. PEDOT, as well as other conductive polymers, was used in several works in combination with electrical stimulation to enhance cell alignment and differentiation. It has been reported the design of an electrical stimulation device using PEDOT:polystyrene-sulfonate (PSS) which was inkjet printed onto a gelatin substrate to guide myotubes alignment and enhance their differentiation. The authors suggested that having a higher content of conductive polymer offered a lower resistance to the passage of electrical current and therefore the risk of overheating, which could damage the cells, was lower [190]. Furthermore, PEDOT:PSS was used to elongate human neural stem cells through the application of pulsed current impacting on their differentiation towards neurons and contributing to longer neurites [191].

Piezoelectric polymers are a class of materials that when are subjected to mechanical stress acquire an electrical polarization evidenced by a difference of potential and electrical charges on their surface. The inverse phenomenon can also be observed where the material can be deformed under the action of an electric field. Among these polymers polyvinylidene fluoride (PVDF) has been widely used for tissue engineering, for instance, in the treatment of neurodegenerative diseases. As an alternative to conventional treatments with neurotrophins, Hoop and co-workers have developed a PVDF membrane to promote neuronal differentiation after stimulation with ultrasound as a non-invasive, spatially precise, and long-term therapy. They observed a differentiation efficiency comparable to conventional in vitro differentiation protocols using the nerve growth factor with a neurite outgrowth uniform in all directions, probably because the piezoelectric stimulation could activate calcium channels in cells, resulting in an increased cellular Ca^2+^ content and thus initiating the adenylyl cyclase (AC) pathway [192]. Ultrasound was also applied to modulate the drug delivery of an anti-restenotic drug from piezoelectric materials. In this case, the authors fabricated an ultra-thin polymeric film for the local treatment of restenosis composed of a poly (lactic acid) supporting membrane on which many polyelectrolyte bilayers containing the drug were deposited. They also added barium titanate nanoparticles with piezoelectric properties in the film and investigated the influence of ultrasound stimuli on the drug release [193].

**Figure 10:**
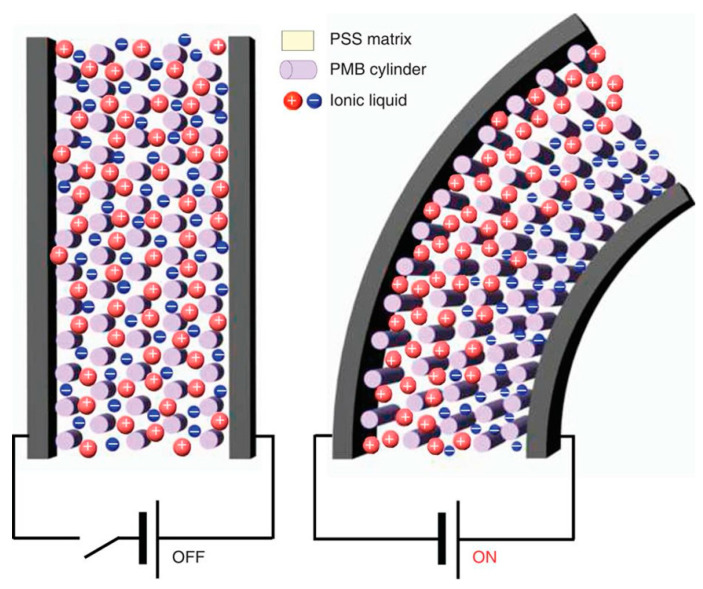
Schematic illustration of the internal structure of the actuator as a result of actuation. The creation of dimensional gradients within the polymer layer was responsible for the fast and efficient electromechanical deformation of the actuator. Reprinted with permission from [194]. Copyright (2013) Nature Publishing Group.

Moreover, electroactive actuators show reversible mechanical deformation in response to electric fields and have received great attention in the area of robotics, microsensors, and artificial muscles. Particularly, ionic polymer actuators were formed by a layer of polymer electrolyte sandwiched in between electrodes and the electromechanical motion in these ionic polymer actuators occurred through the migration of ions towards oppositely charged electrodes upon the application of an electric field. Actuators consisting of single-walled carbon nanotube (SWCNT) electrodes and self-assembled sulphonated block copolymers with the incorporation of ionic liquids. They focused on the development of low-voltage-driven actuators that could be operable over a long period of time with a small battery and they observed that these actuators exhibited much better performance than previously reported ones in terms of long-term durability, large strains, and fast switching response [194] (Figure 10).

Cardiac patches have also been fabricated by seeding neonatal rat cardiomyocytes onto carbon nanotube (CNT)-gelatin methacrylate (GelMA) hydrogels. In this case, CNT-GelMA films showed strong spontaneous and stimulated synchronous beating and the pumping frequency was precisely controllable by applying an external electric field. These films showed three times higher spontaneous synchronous beating rates and 85% lower excitation threshold compared to those cultured on pristine GelMA hydrogels and also enhanced mechanical properties [195].

In conclusion, these smart polymers, in response to an appropriate chemical or electrochemical stimulus can show different answers like swelling/contraction, bending, change of state, or conductivity which make them excellent candidates in engineering nanomedicines and for the development of biomaterials intended to repair or substitute a damage tissue or organ. Table 4 summarizes key examples of redox-responsive materials.

## 6. Magnetic Responsive Nanomaterials

Magnetic responsive nanomaterials and particularly magnetic nanoparticles (MNPs) have interesting features for applications in various fields as a result of many properties, such as high specific surface area, chemical stability, low intraparticle diffusion rate, high loading capacity, and superparamagnetism [196,197]. In the biomedical field, and specifically in tissue regeneration, these kinds of nanoparticles are useful due to their biocompatibility and long-term stability [198]. Furthermore, they have many advantages in terms of penetration and invasiveness since many materials, especially biological tissues, have a lower absorption capacity for magnetic fields than for other types of stimuli, like electric fields, making it possible to remotely activate an event at a relevant distance from the magnet [199]. 

Magnetic-responsive nanomaterials can be grouped into four types: oxides, coated oxides, metallic, and coated metal materials [200]. The first group includes iron oxides or ferrite nanoparticles, ordered in a crystalline state of maghemite or magnetite, which are the most explored. If their size becomes below 128 nm, they change from having ferro or ferrimagnetic properties to a superparamagnetic state, preventing self-agglomeration. This subclass of particles is known as superparamagnetic iron oxide nanoparticles (SPION) and within them, a subclassification also exists in which if their size is less than 50 nm, they are called ultrasmall superparamagnetic iron oxide nanoparticles (USPION) [201]. 

Since ferrite surfaces are relatively inert, it is necessary to improve its reactivity using a coating, generally composed of silica. This surface coating implies several covalent modifications with different functional groups, using organo-silane molecules. Recently, it was demonstrated that magnetite nanoparticles (Fe_3_O_4_-MNPs) modified with silica by TEOS hydrolysis showed the same spherical distribution and structure than MNPS. Even so, the net saturation magnetization increased as the amount of silica was increased on the magnetite nanoparticles [202,203,204].

In the case of magnetic metallic nanoparticles, these ones have a higher magnetic moment than oxides. However, they are pyrophoric (which is the capability of self-ignition at temperatures above 55 °C), and reactive to oxidizing agents, making it difficult for handling [200]. In this way, to prevent the disadvantages of metallic nanoparticles, their surface can be passivized, making a protective layer through different ways, as for example using surfactant agents, polymers, precious metals, or making a gentle oxidation [196].

Top-down and bottom-up approaches are used routinely to synthesize monodisperse magnetic nanoparticles with excellent stability with shape-controllable sizes ranging from a few up to tens of nanometers [205,206]. The methods included in those approaches can be classified in three groups: physical, chemical, and biological. Among them, chemical methods, which correspond to a bottom-up approach, can be conducted in gas and wet phases. Sol-gel, co-precipitation method, hydrothermal synthesis, thermal decomposition, microemulsion, techniques involving the use of high intensity ultrasound (denominated sonolysis), solvothermal, and electrochemical are the most used and cited methods, and correspond to wet phase methods [207].

**Figure 11:**
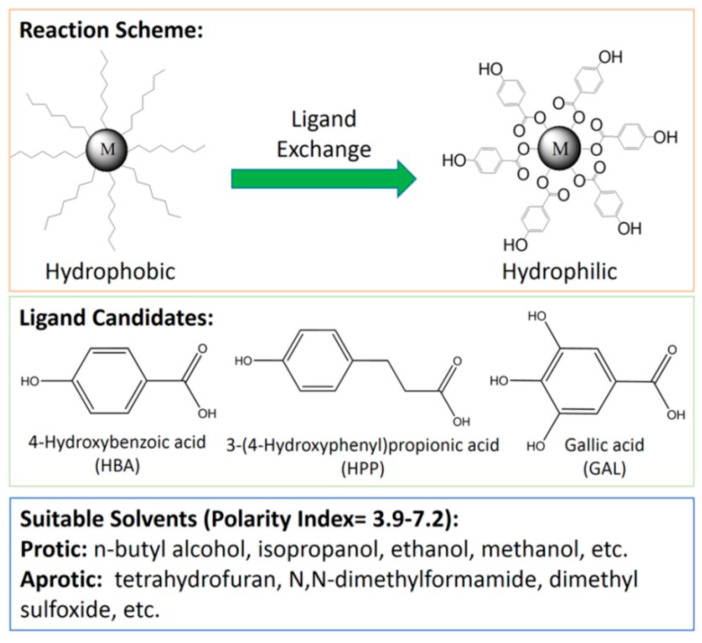
Proposed illustration of one-step ligand exchange reaction, ligand candidates, and the target solvents. Reprinted (adapted) with permission from [214]. Copyright (2014) American Chemical Society.

MNPs can be easily functionalized to be used in several biomedical applications [208,209,210,211,212]. A desired property in biocompatible MNPs is a higher hydrophilicity, thus increasing water solubility [196]. Before surface functionalization, MNPs must be stabilized in nonaqueous solvents using a hydrocarbon layer, and then, such functionalization can be conducted using different methods. The most common ones are ligand addition, ligand exchange, and hydrophilic silica coating. In the ligand addition method, an amphiphilic molecule, that owns a hydrophilic and a hydrophobic group, is used in order to increase the MNPs water solubility [213]. The ligand exchange way is achieved through the use of a new type of coordinating group that links tightly to the surface thanks to chemical bonding and replaces the hydrocarbon layer [214]. Furthermore, this coordinating group has on the other side a polar group, which allows their solubilization in water (Figure 11). The third method, the aforementioned silica coating, is the most adequate to obtain a biocompatible, stable, and hydrophilic property [215]. To achieve this modification, the most common approach is via sol-gel process, including tetraethyl orthosilicate (TEOS) hydrolysis [203].

As previously mentioned, MNPs have been used in several fields [216]. Some of those fields include small molecule drug delivery, hyperthermia therapy, gene therapy, cell tracking, and particularly in tissue engineering [217]. Especially, in bone regeneration, the use of MNPs alone or combined with a magnetic field is particularly beneficial, because three key factors, which are stem cells, growth factors, and scaffolds are improved [218,219]. For example, peptide-MNP conjugates for remote signaling mechano-activation. They demonstrated that remote activation of signaling pathways in mesenchymal cell progenitors using peptide conjugated MNP can regulate remote controlled bone tissue formation [220]. Iron oxide MNPs have also been combined with different materials to potentially be used as a magnetically stimulated system in tissue engineering applications [221]. Combination of MNPs with polysaccharide polymers allows formation of magnetically responsive hydrogels to improve cell proliferation and adhesion in an external magnetic field. This is promising to be used in multiple tissues, for example skin, cartilage, muscle, and connective tissue [222]. In another work, an enzyme-MNP complex was developed to control cell proliferation by an external magnetic field [223]. This switching cell growth system can be potentially used to control cell responses and develop interesting biomaterials for tissue regeneration.

Another important application of superparamagnetic MNPs is to thermally conduct the ablation of pathological cells, or to induce the thermal release of drugs within composites materials, due to the capacity of heating up when they are in proximity of an alternating magnetic field [199,224,225]. Furthermore, some MNPs, in particular SPIONs, are widely used in magnetic resonance imaging (MRI) as contrast agents [216,226,227] or for the treatment of infectious diseases, due their intrinsic activity as antimicrobial agents [228].

The development of magnetic nanoparticle-based therapies for various biomedical applications has been discussed. Although magnetic nanoparticles have been widely used in drug delivery and hyperthermia treatments, recent applications of magnetic nanoparticles have demonstrated their promise towards decreasing implant infection and increasing tissue growth. Table 5 summarizes key examples of magnetic responsive materials.

## 7. Conclusions

Stimuli-responsive materials have a potentially significant future in drug delivery and tissue engineering. These modern materials could deliver therapeutic agents with a controlled and sustained manner. However, there are still several significant challenges to address to uniformly fulfill biosafety and efficacy requirements. Different factors such as material composition and surface modifications, necessary to introduce the responsiveness, have been shown to be important when assessing the biocompatibility and delivery of drugs. In parallel, the protocol employed to develop these materials and the scalability are of paramount importance for the successful implementation of this technology. Even though, while several different stimuli responsive materials and their associated construction methodology are continuously being developed, the potential to reach a successful implementation is excellent.

**Figure 12:**
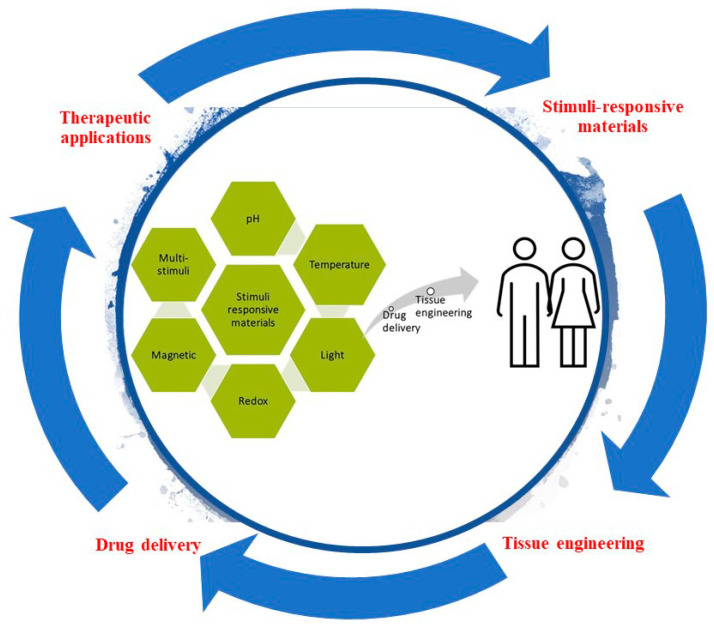
Schematic illustration of therapeutic applications and stimuli responsive materials.

This review highlights some interesting examples from the literature to offer an overview of the most common stimuli-responsive materials relevant to tissue engineering and drug delivery. This emerging field of material science has generated considerable and increasing interest during the past decades. In the particular field of medicinal sciences stimuli-responsive materials offer new opportunities in the treatment of various conditions. The driving force of these and future developments is the huge versality of the field in terms of the materials employed and the stimulus applied (i.e., redox, pH, magnetic, temperature, light) (Figure 12). The criteria for therapeutic applications and stimuli responsive materials properties are not easily met; however, great progress has been made toward this end. We believe that future research will capitalize on opportunities to address the challenges of toxicity posed by some of the components used for stimuli-responsive materials, the development of materials with biomimetic architectural/topological and mechanical properties, and exploration of new stimuli to deliver drugs and instruct cell behavior, perhaps also employing multi-stimuli-responsive materials. 

## Figures and Tables

**Table 1:** List of representative articles employing pH responsive materials.

Stimulus	Material	Drug	Reference
pH	Chitosan hydrogels	Anti-inflammatory factors and antibiotics	[58]
	HEMA (2-hydroxyethyl methacrylate)/DMAEMA (dimethylaminoethyl methacrylate		[59]
	Mesoporous bioglass (MBG) with hydroxyapatite (HAp)	Metformin hydrochloride (MH)	[60]
	Mesoporous ceramics with hydroxypatite (HAp)	Levofloxacin (Levo)	[61]
	Liposomes	Antagomir-10b y Paclitaxel (PTX)	[63,64,65]
	Hydrogels based on cytosine (C) and guanosine (G) modified hyaluronic acid (HA) via hydrogen bonding, with 1,6-hexamethylenediamine (HMDA)		[66]

**Table 2:** List of representative articles employing thermoresponsive materials.

Stimulus	Material	Drug	Reference
Temperature	Biodegradable citrate-based, poly(polyethyleneglycol citrate-co-N-isopropylacrylamide) (PPCN) mixed with gelatin (PPCNG)	BMP9 (growth differentiation factor)	[78]
	1-ethyl-3-(3-dimethyl-aminopropyl)-1-carbodiimide hydrochloride and N-hydroxysuccinimide	Doxorubicin (DOX)	[79]
	Nanoparticles based on gelatin, poly(lactide) and 1,2-dipalmitoyl-sn-glycero-3-phosphoethanolamine (DPPE) (gelatin-co-PLA-DPPE)	Doxorubicin (DOX)	[80]
	Chitosan/β-glycerophosphate	Venlafaxine hydrochloride	[81]
	Chitosan/collagen/β-glycerophosphate		[82]
	Chitosan/β-glycerophosphate/hydroxyapatite		[83]
	Methyl cellulose		[84]
	Methyl cellulose and Collagen		[85]
	Hydroxypropylmethyl cellulose associated with chondroitin-6-sulphate sodium		[86]
	Polypeptides (ELP)		[87]
	poly(N-alkyl substituted acrylamide) PNIPPAm	Doxorubicin (DOX)	[88]
	PNIPPAm with methacrylated hyaluronan		[89]
	PEG and PNIPPAm	DNA	[90]
	Hyaluronic acid-g-poly(N-isopropylacrylamide) and PNIPAAm (AHA-g-PNIPAAm)		[91]
	Chitosan (CS) and poly(ethylene glycol)-poly(N-isopropylacrylamide) (PEGPNIPAAm)	Mesenchymal stem cells (MSCs)	[72]
	Polyisocyanopeptide (PIC)		[93,94]
	Polyurethane dispersion (PU2)	Neural stem cells (NSCs)	[95]
	Methacrylated hyaluronic acid (HAMA), methacrylated poly[N-(2-hydroxypropyl)methacrylamide mono/dilactate] (pHPMAlac)/polyethylene glycol (PEG) and polycaprolactone (PCL)		[96]
	Poly(polyethyleneglycol citrate-co-N-isopropylacrylamide) (PPCN) mixed with gelatin (PPCNG) and Graphene oxide	MSCs with BMP9	[73]
	Poly(N-vinyl caprolactam) (PVCL) with Clay nanocomposite		[97]

**Table 3:** List of representative articles employing light responsive materials.

Stimulus	Material	Drug	Reference
Light	Azobenzene-glycolipid into liposome		[112]
	Liposomes formed by decyl-azobenzyl-triethylammonium and cholesterol sulfate	Hydrophobic content	[113]
	Micelles of polyglycerol using spiropyran	Hydrophobic content	[115,116]
	Polyacrylamide-based hydrogel with azobenzene	Mesenchymal stem cells	[124]
	Colloidal gold encapsulated in liposomes		[132]
	Liposomes with multibranched gold nanoantennas		[129]
	poly(3-hexylthiophene)		[136]
	Micelle from poly(S-(o-nitrobenzyl)-L-cysteine)-b-poly(ethylene glycol) block copolymers		[142]
	The nitrobenzyl group incorporated into liposomes		[143,145]
	Poly(ethylene glycol) (PEG)-based hydrogel		[149]
	PEG and hyaluronic acid (HA)		[157]
	Polyesters hydrogels		[158]

**Table 4:** List of representative articles employing redox-responsive materials.

Stimulus	Material	Drug	Reference
Redox	Glycolipid, chitosan, and stearic acid	Doxorubicin (DOX)	[162]
	Polyethylene glycol and polycaprolactone	Doxorubicin (DOX)	[163]
	Keratin nanoparticles		[165]
	Keratin grafted poly(N-(2-hydroxypropy l)methacrylamide)	Doxorubicin (DOX)	[166]
	Poly(acrylic acid) modified with cyclodextrins (pAA-CDs)		[173]
	pAA with ferrocene (pAA-Fc)		
	Poly (1-vinyl-2-pyrrolidinone) modified with O-carboxymethyl chitosan and acrylamide		[174]
	Ferrocene complexes with cyclodextrins		[175]
	Oligoviologen		[176]
	Viologen-cyclodextrin complexes		[177]
	Polyamidoamine (PAMAM) dendrimers with tetrathiafulvalene (TTF)		[180]
	TTF with poly(NIPAM)		[181]
	Polypyrrole (PPy)	Interleukin IL-3	[188]
	Poly (3,4-ethylenedioxythiophene) (PEDOT) and polystyrene-sulfonate (PSS)		[190,191]
	Polymers polyvinylidene fluoride (PVDF)		[192]
	Poly (lactic acid) and barium titanate nanoparticles		[193]
	Carbon nanotube (SWCNT)		[194]
	Carbon nanotube (CNT)-gelatin methacrylate (GelMA)		[195]

**Table 5:** List of representative articles employing magnetic responsive materials.

Stimulus	Material	Drug	Reference
Magnetic	Peptide-MNPs (magnetic nanoparticles)		[220]
	Iron oxide MNPs		[221]
	MNPs with polysaccharide-based polymers		[222]
	Enzyme-MNP complex		[223]
	Iron oxide nanoparticles (USPION)		[216,226,227]

## References

[1] Lumelsky N., O’Hayre M., Chander P., Shum L., Somerman M.J. (2018). Autotherapies: Enhancing Endogenous Healing and Regeneration. Trends Mol. Med..

[2] Lavrador P., Gaspar V.M., Mano J.F. (2018). Stimuli-responsive nanocarriers for delivery of bone therapeutics–Barriers and progresses. J. Control. Release.

[3] Rogina A., Ressler A., Matić I., Gallego Ferrer G., Marijanović I., Ivanković M., Ivanković H. (2017). Cellular hydrogels based on pH-responsive chitosan-hydroxyapatite system. Carbohydr. Polym..

[4] Echazú M.I.A., Olivetti C.E., Peralta I., Alonso M.R., Anesini C., Perez C.J., Alvarez G.S., Desimone M.F. (2018). Development of pH-responsive biopolymer-silica composites loaded with Larrea divaricata Cav. extract with antioxidant activity. Colloids Surfaces B Biointerfaces.

[5] Parani M., Lokhande G., Singh A., Gaharwar A.K. (2016). Engineered Nanomaterials for Infection Control and Healing Acute and Chronic Wounds. ACS Appl. Mater. Interfaces.

[6] Hamdan S., Pastar I., Drakulich S., Dikici E., Tomic-Canic M., Deo S., Daunert S. (2017). Nanotechnology-Driven Therapeutic Interventions in Wound Healing: Potential Uses and Applications. ACS Cent. Sci..

[7] Bose S., Robertson S.F., Bandyopadhyay A. (2018). Surface modification of biomaterials and biomedical devices using additive manufacturing. Acta Biomater..

[8] Wu G., Li P., Feng H., Zhang X., Chu P.K. (2015). Engineering and functionalization of biomaterials via surface modification. J. Mater. Chem. B.

[9] Pezzoni M., Catalano P.N., Pizarro R.A., Desimone M.F., Soler-Illia G.J.A.A., Bellino M.G., Costa C.S. (2017). Antibiofilm effect of supramolecularly templated mesoporous silica coatings. Mater. Sci. Eng. C.

[10] Catalano P.N., Pezzoni M., Costa C., Soler-Illia G.J.D.A.A., Bellino M.G., Desimone M.F. (2016). Optically transparent silver-loaded mesoporous thin film coating with long-lasting antibacterial activity. Microporous Mesoporous Mater..

[11] Bellino M.G., Golbert S., De Marzi M.C., Soler-Illia G.J.A.A., Desimone M.F. (2013). Controlled adhesion and proliferation of a human osteoblastic cell line by tuning the nanoporosity of titania and silica coatings. Biomater. Sci..

[12] Badeau B.A., DeForest C.A. (2019). Programming Stimuli-Responsive Behavior into Biomaterials. Annu. Rev. Biomed. Eng..

[13] Ooi H.W., Hafeez S., van Blitterswijk C.A., Moroni L., Baker M.B. (2017). Hydrogels that listen to cells: A review of cell-responsive strategies in biomaterial design for tissue regeneration. Mater. Horizons.

[14] Kondiah P., Choonara Y., Kondiah P., Marimuthu T., Kumar P., du Toit L., Pillay V. (2016). A Review of Injectable Polymeric Hydrogel Systems for Application in Bone Tissue Engineering. Molecules.

[15] Albert K., Hsu H.-Y. (2016). Carbon-Based Materials for Photo-Triggered Theranostic Applications. Molecules.

[16] Shen L. (2011). Biocompatible Polymer/Quantum Dots Hybrid Materials: Current Status and Future Developments. J. Funct. Biomater..

[17] Galdopórpora J.M., Morcillo M.F., Ibar A., Perez C.J., Tuttolomondo M.V., Desimone M.F. (2019). Development of Silver Nanoparticles/Gelatin Thermoresponsive Nanocomposites: Characterization and Antimicrobial Activity. Curr. Pharm. Des..

[18] Mousavi S.T., Harper G.R., Municoy S., Ashton M.D., Townsend D., Alsharif G.H.K., Oikonomou V.K., Firlak M., Au-Yong S., Murdock B.E. (2020). Electroactive Silk Fibroin Films for Electrochemically Enhanced Delivery of Drugs. Macromol. Mater. Eng..

[19] Gonçalves G.A.R., Paiva R.D.M.A. (2017). Gene therapy: Advances, challenges and perspectives. Einstein (São Paulo).

[20] Pattni B.S., Torchilin V.P., Devarajan P.V., Jain S. (2015). Targeted Drug Delivery Systems: Strategies and Challenges. Targeted Drug Delivery: Concepts and Design.

[21] Khademhosseini A., Langer R. (2016). A decade of progress in tissue engineering. Nat. Protoc..

[22] Mura S., Nicolas J., Couvreur P. (2013). Stimuli-responsive nanocarriers for drug delivery. Nat. Mater..

[23] Gracia R., Mecerreyes D. (2013). Polymers with redox properties: Materials for batteries, biosensors and more. Polym. Chem..

[24] Huo M., Yuan J., Tao L., Wei Y. (2014). Redox-responsive polymers for drug delivery: from molecular design to applications. Polym. Chem..

[25] Hardy J.G., Lee J.Y., Schmidt C.E. (2013). Biomimetic conducting polymer-based tissue scaffolds. Curr. Opin. Biotechnol..

[26] Rajabi A.H., Jaffe M., Arinzeh T.L. (2015). Piezoelectric materials for tissue regeneration: A review. Acta Biomater..

[27] Baxter F.R., Bowen C.R., Turner I.G., Dent A.C.E. (2010). Electrically active bioceramics: A review of interfacial responses. Ann. Biomed. Eng..

[28] Ribeiro C., Sencadas V., Correia D.M., Lanceros-Méndez S. (2015). Piezoelectric polymers as biomaterials for tissue engineering applications. Colloids Surfaces B Biointerfaces.

[29] Chorsi M.T., Curry E.J., Chorsi H.T., Das R., Baroody J., Purohit P.K., Ilies H., Nguyen T.D. (2018). Piezoelectric Biomaterials for Sensors and Actuators. Adv. Mater..

[30] Yuan H., Lei T., Qin Y., He J.H., Yang R. (2019). Design and application of piezoelectric biomaterials. J. Phys. D. Appl. Phys..

[31] Kapat K., Shubhra Q.T.H., Zhou M., Leeuwenburgh S. (2020). Piezoelectric Nano-Biomaterials for Biomedicine and Tissue Regeneration. Adv. Funct. Mater..

[32] Kocak G., Tuncer C., Bütün V. (2017). pH-Responsive polymers. Polym. Chem..

[33] Omidi M., Yadegari A., Tayebi L. (2017). Wound dressing application of pH-sensitive carbon dots/chitosan hydrogel. RSC Adv..

[34] Banerjee I., Mishra D., Das T., Maiti T.K. (2012). Wound pH-Responsive Sustained Release of Therapeutics from a Poly(NIPAAm-co-AAc) Hydrogel. J. Biomater. Sci. Polym. Ed..

[35] Ninan N., Forget A., Shastri V.P., Voelcker N.H., Blencowe A. (2016). Antibacterial and Anti-Inflammatory pH-Responsive Tannic Acid-Carboxylated Agarose Composite Hydrogels for Wound Healing. ACS Appl. Mater. Interfaces.

[36] Qiao Y., Wan J., Zhou L., Ma W., Yang Y., Luo W., Yu Z., Wang H. (2019). Stimuli-responsive nanotherapeutics for precision drug delivery and cancer therapy. WIREs Nanomed. Nanobiotechnol..

[37] Ferreira N.N., Ferreira L.M.B., Cardoso V.M.O., Boni F.I., Souza A.L.R., Gremião M.P.D. (2018). Recent advances in smart hydrogels for biomedical applications: From self-assembly to functional approaches. Eur. Polym. J..

[38] Karimi M., Eslami M., Sahandi-Zangabad P., Mirab F., Farajisafiloo N., Shafaei Z., Ghosh D., Bozorgomid M., Dashkhaneh F., Hamblin M.R. (2016). pH-Sensitive stimulus-responsive nanocarriers for targeted delivery of therapeutic agents. WIREs Nanomed. Nanobiotechnol..

[39] Adedoyin A.A., Ekenseair A.K. (2018). Biomedical applications of magneto-responsive scaffolds. Nano Res..

[40] Katz J.S., Burdick J.A. (2010). Light-responsive biomaterials: Development and applications. Macromol. Biosci..

[41] Ward M.A., Georgiou T.K. (2011). Thermoresponsive Polymers for Biomedical Applications. Polymers.

[42] Sponchioni M., Capasso Palmiero U., Moscatelli D. (2019). Thermo-responsive polymers: Applications of smart materials in drug delivery and tissue engineering. Mater. Sci. Eng. C.

[43] Zarrintaj P., Jouyandeh M., Ganjali M.R., Hadavand B.S., Mozafari M., Sheiko S.S., Vatankhah-Varnoosfaderani M., Gutiérrez T.J., Saeb M.R. (2019). Thermo-sensitive polymers in medicine: A review. Eur. Polym. J..

[44] Zhang J., Jiang X., Wen X., Xu Q., Zeng H., Zhao Y., Liu M., Wang Z., Hu X., Wang Y. (2019). Bio-responsive smart polymers and biomedical applications. J. Phys. Mater..

[45] Fu X., Hosta-Rigau L., Chandrawati R., Cui J. (2018). Multi-Stimuli-Responsive Polymer Particles, Films, and Hydrogels for Drug Delivery. Chem.

[46] Hajebi S., Rabiee N., Bagherzadeh M., Ahmadi S., Rabiee M., Roghani-Mamaqani H., Tahriri M., Tayebi L., Hamblin M.R. (2019). Stimulus-responsive polymeric nanogels as smart drug delivery systems. Acta Biomater..

[47] Sood N., Bhardwaj A., Mehta S., Mehta A. (2016). Stimuli-responsive hydrogels in drug delivery and tissue engineering. Drug Deliv..

[48] Koetting M.C., Peters J.T., Steichen S.D., Peppas N.A. (2015). Stimulus-responsive hydrogels: Theory, modern advances, and applications. Mater. Sci. Eng. R Reports.

[49] Shah A., Malik M.S., Khan G.S., Nosheen E., Iftikhar F.J., Khan F.A., Shukla S.S., Akhter M.S., Kraatz H.-B., Aminabhavi T.M. (2018). Stimuli-responsive peptide-based biomaterials as drug delivery systems. Chem. Eng. J..

[50] Khan F., Tanaka M. (2018). Designing Smart Biomaterials for Tissue Engineering. Int. J. Mol. Sci..

[51] Webb B.A., Chimenti M., Jacobson M.P., Barber D.L. (2011). Dysregulated pH: A perfect storm for cancer progression. Nat. Rev. Cancer.

[52] Sahle F.F., Gulfam M., Lowe T.L. (2018). Design strategies for physical-stimuli-responsive programmable nanotherapeutics. Drug Discov. Today.

[53] Hu Y.B., Dammer E.B., Ren R.J., Wang G. (2015). The endosomal-lysosomal system: From acidification and cargo sorting to neurodegeneration. Transl. Neurodegener..

[54] Wang L., Neumann M., Fu T., Li W., Cheng X., Su B.-L. (2018). Porous and responsive hydrogels for cell therapy. Curr. Opin. Colloid Interface Sci..

[55] Perez R.A., Singh R.K., Kim T.-H., Kim H.-W. (2017). Silica-based multifunctional nanodelivery systems toward regenerative medicine. Mater. Horizons.

[56] Wang Y., Byrne J.D., Napier M.E., DeSimone J.M. (2012). Engineering nanomedicines using stimuli-responsive biomaterials. Adv. Drug Deliv. Rev..

[57] Schneider L.A., Korber A., Grabbe S., Dissemond J. (2007). Influence of pH on wound-healing: A new perspective for wound-therapy?. Arch. Dermatol. Res..

[58] Zhu L., Bratlie K.M. (2018). pH sensitive methacrylated chitosan hydrogels with tunable physical and chemical properties. Biochem. Eng. J..

[59] You J.-O., Rafat M., Almeda D., Maldonado N., Guo P., Nabzdyk C.S., Chun M., LoGerfo F.W., Hutchinson J.W., Pradhan-Nabzdyk L.K. (2015). pH-responsive scaffolds generate a pro-healing response. Biomaterials.

[60] Yang C., Guo W., Cui L., Xiang D., Cai K., Lin H., Qu F. (2014). pH-responsive controlled-release system based on mesoporous bioglass materials capped with mineralized hydroxyapatite. Mater. Sci. Eng. C.

[61] Cicuéndez M., Doadrio J.C., Hernández A., Portolés M.T., Izquierdo-Barba I., Vallet-Regí M. (2018). Multifunctional pH sensitive 3D scaffolds for treatment and prevention of bone infection. Acta Biomater..

[62] Gulzar A., Gai S., Yang P., Li C., Ansari M.B., Lin J. (2015). Stimuli responsive drug delivery application of polymer and silica in biomedicine. J. Mater. Chem. B.

[63] Lennox K.A., Owczarzy R., Thomas D.M., Walder J.A., Behlke M.A. (2013). Improved Performance of Anti-miRNA Oligonucleotides Using a Novel Non-Nucleotide Modifier. Mol. Ther. Nucleic Acids.

[64] Makovitzki A., Fink A., Shai Y. (2009). Suppression of human solid tumor growth inmice by intratumor and systemic inoculation of histidine-rich and pH-dependent host defense–like lytic peptides. Cancer Res..

[65] Zhang Q., Ran R., Zhang L., Liu Y., Mei L., Zhang Z., Gao H., He Q. (2015). Simultaneous delivery of therapeutic antagomirs with paclitaxel for the management of metastatic tumors by a pH-responsive anti-microbial peptide-mediated liposomal delivery system. J. Control. Release.

[66] Ye X., Li X., Shen Y., Chang G., Yang J., Gu Z. (2017). Self-healing pH-sensitive cytosine- and guanosine-modified hyaluronic acid hydrogels via hydrogen bonding. Polymer.

[67] Han D., Steckl A.J. (2017). Selective pH-Responsive Core–Sheath Nanofiber Membranes for Chem/Bio/Med Applications: Targeted Delivery of Functional Molecules. ACS Appl. Mater. Interfaces.

[68] Tagami T., Foltz W.D., Ernsting M.J., Lee C.M., Tannock I.F., May J.P., Li S.-D. (2011). MRI monitoring of intratumoral drug delivery and prediction of the therapeutic effect with a multifunctional thermosensitive liposome. Biomaterials.

[69] Yang H.Y., Li Y., Lee D.S. (2018). Multifunctional and Stimuli-Responsive Magnetic Nanoparticle-Based Delivery Systems for Biomedical Applications. Adv. Ther..

[70] Miguel S.P., Ribeiro M.P., Brancal H., Coutinho P., Correia I.J. (2014). Thermoresponsive chitosan–agarose hydrogel for skin regeneration. Carbohydr. Polym..

[71] Katas H., Wen C.Y., Siddique M.I., Hussain Z., Mohd Fadhil F.H. (2017). Thermoresponsive curcumin/DsiRNA nanoparticle gels for the treatment of diabetic wounds: Synthesis and drug release. Ther. Deliv..

[72] Mellati A., Fan C.-M., Tamayol A., Annabi N., Dai S., Bi J., Jin B., Xian C., Khademhosseini A., Zhang H. (2016). Microengineered 3D cell-laden thermoresponsive hydrogels for mimicking cell morphology and orientation in cartilage tissue engineering. Biotechnol. Bioeng..

[73] Zhao C., Zeng Z., Qazvini N.T., Yu X., Zhang R., Yan S., Shu Y., Zhu Y., Duan C., Bishop E. (2018). Thermoresponsive Citrate-Based Graphene Oxide Scaffold Enhances Bone Regeneration from BMP9-Stimulated Adipose-Derived Mesenchymal Stem Cells. ACS Biomater. Sci. Eng..

[74] Meewes M., Ricka J., De Silva M., Nyffenegger R., Binkert T. (1991). Coil-globule transition of poly(N-isopropylacrylamide): A study of surfactant effects by light scattering. Macromolecules.

[75] Zhu Y., Batchelor R., Lowe A.B., Roth P.J. (2016). Design of Thermoresponsive Polymers with Aqueous LCST, UCST, or Both: Modification of a Reactive Poly(2-vinyl-4,4-dimethylazlactone) Scaffold. Macromolecules.

[76] Mah E., Ghosh R., Mah E., Ghosh R. (2013). Thermo-Responsive Hydrogels for Stimuli-Responsive Membranes. Processes.

[77] Vanparijs N., Nuhn L., De Geest B.G. (2017). Transiently thermoresponsive polymers and their applications in biomedicine. Chem. Soc. Rev..

[78] Ye J., Wang J., Zhu Y., Wei Q., Wang X., Yang J., Tang S., Liu H., Fan J., Zhang F. (2016). A thermoresponsive polydiolcitrate-gelatin scaffold and delivery system mediates effective bone formation from BMP9-transduced mesenchymal stem cells. Biomed. Mater..

[79] Slemming-Adamsen P., Song J., Dong M., Besenbacher F., Chen M. (2015). In Situ Cross-Linked PNIPAM/Gelatin Nanofibers for Thermo-Responsive Drug Release. Macromol. Mater. Eng..

[80] Han S., Li M., Liu X., Gao H., Wu Y. (2013). Construction of amphiphilic copolymer nanoparticles based on gelatin as drug carriers for doxorubicin delivery. Colloids Surfaces B Biointerfaces.

[81] Peng Y., Li J., Li J., Fei Y., Dong J., Pan W. (2013). Optimization of thermosensitive chitosan hydrogels for the sustained delivery of venlafaxine hydrochloride. Int. J. Pharm..

[82] Dang Q., Liu K., Zhang Z., Liu C., Liu X., Xin Y., Cheng X., Xu T., Cha D., Fan B. (2017). Fabrication and evaluation of thermosensitive chitosan/collagen/α, β-glycerophosphate hydrogels for tissue regeneration. Carbohydr. Polym..

[83] Chen Y., Zhang F., Fu Q., Liu Y., Wang Z., Qi N. (2016). In vitro proliferation and osteogenic differentiation of human dental pulp stem cells in injectable thermo-sensitive chitosan/β-glycerophosphate/hydroxyapatite hydrogel. J. Biomater. Appl..

[84] Cochis A., Bonetti L., Sorrentino R., Contessi Negrini N., Grassi F., Leigheb M., Rimondini L., Farè S. (2018). 3D Printing of Thermo-Responsive Methylcellulose Hydrogels for Cell-Sheet Engineering. Materials.

[85] Thirumala S., Gimble J., Devireddy R., Thirumala S., Gimble J.M., Devireddy R.V. (2013). Methylcellulose Based Thermally Reversible Hydrogel System for Tissue Engineering Applications. Cells.

[86] Sandri G., Bonferoni M.C., Rossi S., Ferrari F., Mori M., Del Fante C., Perotti C., Caramella C. (2012). Thermosensitive eyedrops containing platelet lysate for the treatment of corneal ulcers. Int. J. Pharm..

[87] McDaniel J.R., Callahan D.J., Chilkoti A. (2010). Drug delivery to solid tumors by elastin-like polypeptides. Adv. Drug Deliv. Rev..

[88] Li J., Zha Z., Ge Z., Candiani G. (2016). Thermo-Responsive Polyplex Micelles with PEG Shells and PNIPAM Layer to Protect DNA Cores for Systemic Gene Therapy. Non-Viral Gene Delivery Vectors: Methods and Protocols.

[89] Purushotham S., Chang P.E.J., Rumpel H., Kee I.H.C., Ng R.T.H., Chow P.K.H., Tan C.K., Ramanujan R. (2009). V Thermoresponsive core-shell magnetic nanoparticles for combined modalities of cancer therapy. Nanotechnology.

[90] Kesti M., Müller M., Becher J., Schnabelrauch M., D’Este M., Eglin D., Zenobi-Wong M. (2015). A versatile bioink for three-dimensional printing of cellular scaffolds based on thermally and photo-triggered tandem gelation. Acta Biomater..

[91] Tan H., Ramirez C.M., Miljkovic N., Li H., Rubin J.P., Marra K.G. (2009). Thermosensitive injectable hyaluronic acid hydrogel for adipose tissue engineering. Biomaterials.

[92] Brunelle A.R., Horner C.B., Low K., Ico G., Nam J. (2018). Electrospun thermosensitive hydrogel scaffold for enhanced chondrogenesis of human mesenchymal stem cells. Acta Biomater..

[93] op’t Veld R.C., van den Boomen O.I., Lundvig D.M.S., Bronkhorst E.M., Kouwer P.H.J., Jansen J.A., Middelkoop E., Von den Hoff J.W., Rowan A.E., Wagener F.A.D.T.G. (2018). Thermosensitive biomimetic polyisocyanopeptide hydrogels may facilitate wound repair. Biomaterials.

[94] Zimoch J., Padial J.S., Klar A.S., Vallmajo-Martin Q., Meuli M., Biedermann T., Wilson C.J., Rowan A., Reichmann E. (2018). Polyisocyanopeptide hydrogels: A novel thermo-responsive hydrogel supporting pre-vascularization and the development of organotypic structures. Acta Biomater..

[95] Hsieh F.-Y., Lin H.-H., Hsu S. (2015). 3D bioprinting of neural stem cell-laden thermoresponsive biodegradable polyurethane hydrogel and potential in central nervous system repair. Biomaterials.

[96] Mouser V.H.M., Abbadessa A., Levato R., Hennink W.E., Vermonden T., Gawlitta D., Malda J. (2017). Development of a thermosensitive HAMA-containing bio-ink for the fabrication of composite cartilage repair constructs. Biofabrication.

[97] Shi K., Liu Z., Yang C., Li X.-Y., Sun Y.-M., Deng Y., Wang W., Ju X.-J., Xie R., Chu L.-Y. (2017). Novel Biocompatible Thermoresponsive Poly(N-vinyl Caprolactam)/Clay Nanocomposite Hydrogels with Macroporous Structure and Improved Mechanical Characteristics. ACS Appl. Mater. Interfaces.

[98] Bhullar S.K., Lala N.L., Ramkrishna S. (2015). Smart Biomaterials—A review. Rev. Adv. Mater. Sci.

[99] Ruskowitz E.R., Deforest C.A. (2018). Photoresponsive biomaterials for targeted drug delivery and 4D cell culture. Nat. Rev. Mater..

[100] Leung S.J., Romanowski M. (2012). Light-activated content release from liposomes. Theranostics.

[101] Jerca F.A., Jerca V., Stancu I.-C. (2018). Development and Characterization of Photoresponsive Polymers. Polymer and Photonic Materials Towards Biomedical Breakthroughs.

[102] Ercole F., Davis T.P., Evans R.A. (2010). Photo-responsive systems and biomaterials: Photochromic polymers, light-triggered self-assembly, surface modification, fluorescence modulation and beyond. Polym. Chem..

[103] Wu S., Butt H.-J. (2016). Near-Infrared-Sensitive Materials Based on Upconverting Nanoparticles. Adv. Mater..

[104] Linsley C.S., Wu B.M. (2017). Recent advances in light-responsive on- demand drug-delivery systems. Ther. Deliv.

[105] Bandarab H.M.D., Burdette S.C. (2012). Photoisomerization in different classes of azobenzene. Chem. Soc. Rev..

[106] Udayabhaskararao T., Kundu P.K., Ahrens J., Klajn R. (2015). Reversible Photoisomerization of Spiropyran on the Surfaces of Au25 Nanoclusters. ChemPhysChem.

[107] Ahmad Z., Shah A., Siddiq M., Kraatz H.B. (2014). Polymeric micelles as drug delivery vehicles. RSC Adv..

[108] Madaan K., Kumar S., Poonia N., Lather V., Pandita D. (2014). Dendrimers in drug delivery and targeting: Drug-dendrimer interactions and toxicity issues. J. Pharm. Bioallied Sci..

[109] Alavi M., Karimi N., Safaei M. (2017). Application of various types of liposomes in drug delivery systems. Adv. Pharm. Bull..

[110] Urban P., Pritzl S.D., Konrad D.B., Frank J.A., Pernpeintner C., Roeske C.R., Trauner D., Lohmüller T. (2018). Light-Controlled Lipid Interaction and Membrane Organization in Photolipid Bilayer Vesicles. Langmuir.

[111] Yao C., Wang P., Li X., Hu X., Hou J., Wang L., Zhang F. (2016). Near-Infrared-Triggered Azobenzene-Liposome/Upconversion Nanoparticle Hybrid Vesicles for Remotely Controlled Drug Delivery to Overcome Cancer Multidrug Resistance. Adv. Mater..

[112] Liu D., Wang S., Xu S., Liu H. (2017). Photocontrollable intermittent release of doxorubicin hydrochloride from liposomes embedded by azobenzene-contained glycolipid. Langmuir.

[113] Cui Z.-K., Phoeung T., Rousseau P.-A., Rydzek G., Zhang Q., Bazuin C.G., Lafleur M. (2014). Nonphospholipid Fluid Liposomes with Switchable Photocontrolled Release. Langmuir.

[114] Pearson S., Vitucci D., Khine Y.Y., Dag A., Lu H., Save M., Billon L., Stenzel M.H. (2015). Light-responsive azobenzene-based glycopolymer micelles for targeted drug delivery to melanoma cells. Eur. Polym. J..

[115] Son S., Shin E., Kim B.S. (2014). Light-responsive micelles of spiropyran initiated hyperbranched polyglycerol for smart drug delivery. Biomacromolecules.

[116] Petriashvili G., Devadze L., Zurabishvili T., Sepashvili N., Chubinidze K. (2016). Light controlled drug delivery containers based on spiropyran doped liquid crystal micro spheres. Biomed. Opt. Express.

[117] Baroli B. (2006). Photopolymerization of biomaterials: Issues and potentialities in drug delivery, tissue engineering, and cell encapsulation applications. J. Chem. Technol. Biotechnol..

[118] Nguyen K.T., West J.L. (2002). Photopolymerizable hydrogels for tissue engineering applications. Biomaterials.

[119] Cao Z., Bian Q., Chen Y., Liang F., Wang G. (2017). Light-Responsive Janus-Particle-Based Coatings for Cell Capture and Release. ACS Macro Lett..

[120] Yu L., Schlaich C., Hou Y., Zhang J., Noeske P.-L.M., Haag R. (2018). Photoregulating Antifouling and Bioadhesion Functional Coating Surface Based on Spiropyran. Chem. A Eur. J..

[121] Fedele C., Netti P.A., Cavalli S. (2018). Azobenzene-based polymers: Emerging applications as cell culture platforms. Biomater. Sci..

[122] Shi P., Ju E., Yan Z., Gao N., Wang J., Hou J., Zhang Y., Ren J., Qu X. (2016). Spatiotemporal control of cell-cell reversible interactions using molecular engineering. Nat. Commun..

[123] Pennacchio F.A., Fedele C., De Martino S., Cavalli S., Vecchione R., Netti P.A. (2018). Three-Dimensional Microstructured Azobenzene-Containing Gelatin as a Photoactuable Cell Confining System. ACS Appl. Mater. Interfaces.

[124] Lee I.N., Dobre O., Richards D., Ballestrem C., Curran J.M., Hunt J.A., Richardson S.M., Swift J., Wong L.S. (2018). Photoresponsive Hydrogels with Photoswitchable Mechanical Properties Allow Time-Resolved Analysis of Cellular Responses to Matrix Stiffening. ACS Appl. Mater. Interfaces.

[125] O’Brien P., Thomas P.J. (2013). Nanoscience.

[126] Chen Y., Li H., Deng Y., Sun H., Ke X., Ci T. (2017). Near-infrared light triggered drug delivery system for higher efficacy of combined chemo-photothermal treatment. Acta Biomater..

[127] Guha S., Shaw S.K., Spence G.T., Roland F.M., Smith B.D. (2015). Clean Photothermal Heating and Controlled Release from Near-Infrared Dye Doped Nanoparticles without Oxygen Photosensitization. Langmuir.

[128] Bao Z., Liu X., Liu Y., Liu H., Zhao K. (2016). Near-infrared light-responsive inorganic nanomaterials for photothermal therapy. Asian J. Pharm. Sci..

[129] Ou Y.-C., Webb J.A., Faley S., Shae D., Talbert E.M., Lin S., Cutright C.C., Wilson J.T., Bellan L.M., Bardhan R. (2016). Gold Nanoantenna-Mediated Photothermal Drug Delivery from Thermosensitive Liposomes in Breast Cancer. ACS omega.

[130] Guerrero A.R., Hassan N., Escobar C.A., Albericio F., Kogan M.J., Araya E. (2014). Gold nanoparticles for photothermally controlled drug release. Nanomedicine.

[131] Alkilany A.M., Thompson L.B., Boulos S.P., Sisco P.N., Murphy C.J. (2012). Gold nanorods: Their potential for photothermal therapeutics and drug delivery, tempered by the complexity of their biological interactions. Adv. Drug Deliv. Rev..

[132] Mathiyazhakan M., Wiraja C., Xu C. (2018). A Concise Review of Gold Nanoparticles-Based Photo-Responsive Liposomes for Controlled Drug Delivery. Nano-Micro Lett..

[133] Norouzi H., Khoshgard K., Akbarzadeh F. (2018). In vitro outlook of gold nanoparticles in photo-thermal therapy: a literature review. Lasers Med. Sci..

[134] Lajunen T., Viitala L., Kontturi L.S., Laaksonen T., Liang H., Vuorimaa-Laukkanen E., Viitala T., Le Guével X., Yliperttula M., Murtomäki L. (2015). Light induced cytosolic drug delivery from liposomes with gold nanoparticles. J. Control. Release.

[135] Xing R., Liu K., Jiao T., Zhang N., Ma K., Zhang R., Zou Q., Ma G., Yan X. (2016). An Injectable Self-Assembling Collagen-Gold Hybrid Hydrogel for Combinatorial Antitumor Photothermal/Photodynamic Therapy. Adv. Mater..

[136] Martino N., Feyen P., Porro M., Bossio C., Zucchetti E., Ghezzi D., Benfenati F., Lanzani G., Antognazza M.R. (2015). Photothermal cellular stimulation in functional bio-polymer interfaces. Sci. Rep..

[137] Bao C., Zhu L., Lin Q., Tian H. (2015). Building biomedical materials using photochemical bond cleavage. Adv. Mater..

[138] Barhoumi A., Liu Q., Kohane D.S. (2015). Ultraviolet light-mediated drug delivery: Principles, applications, and challenges. J. Control. Release.

[139] Chin A.L., Zhong Y., Tong R. (2017). Emerging strategies in near-infrared light triggered drug delivery using organic nanomaterials. Biomater. Sci..

[140] Yan B., Boyer J.-C., Branda N.R., Zhao Y. (2011). Near-Infrared Light-Triggered Dissociation of Block Copolymer Micelles Using Upconverting Nanoparticles. J. Am. Chem. Soc..

[141] Klán P., Šolomek T., Bochet C.G., Blanc A., Givens R., Rubina M., Popik V., Kostikov A., Wirz J. (2013). Photoremovable protecting groups in chemistry and biology: Reaction mechanisms and efficacy. Chem. Rev..

[142] Liu G., Dong C.-M.M. (2012). Photoresponsive Poly(S-(o-nitrobenzyl)-l-cysteine)-b-PEO from a l-Cysteine N-Carboxyanhydride Monomer: Synthesis, Self-Assembly, and Phototriggered Drug Release. Biomacromolecules.

[143] Zhang D., Liu Z., Konetski D., Wang C., Worrell B.T., Bowman C.N. (2018). Liposomes formed from photo-cleavable phospholipids:: In situ formation and photo-induced enhancement in permeability. RSC Adv..

[144] Chandra B., Subramaniam R., Mallik S., Srivastava D.K. (2006). Formulation of photocleavable liposomes and the mechanism of their content release. Org. Biomol. Chem..

[145] Bayer A.M., Alam S., Mattern-Schain S.I., Best M.D. (2014). Triggered liposomal release through a synthetic phosphatidylcholine analogue bearing a photocleavable moiety embedded within the sn-2 acyl chain. Chem. A Eur. J..

[146] Griffin D.R., Kasko A.M. (2012). Photodegradable macromers and hydrogels for live cell encapsulation and release. J Am Chem Soc..

[147] Liu H., Song Z., Chen X. (2014). Photo-controllable molecular hydrogels for drug delivery. J. Nanosci. Nanotechnol..

[148] Kloxin A.M., Kasko A.M., Salinas C.N., Anseth K.S. (2009). Photodegradable hydrogels for dynamic tuning of physical and chemical properties. Science.

[149] Azagarsamy M.A., McKinnon D.D., Alge D.L., Anseth K.S. (2014). Coumarin-based photodegradable hydrogel: Design, synthesis, gelation, and degradation kinetics. ACS Macro Lett..

[150] De Gracia Lux C., Lux J., Collet G., He S., Chan M., Olejniczak J., Foucault-Collet A., Almutairi A. (2015). Short Soluble Coumarin Crosslinkers for Light-Controlled Release of Cells and Proteins from Hydrogels. Biomacromolecules.

[151] Kim S.H., Sun Y., Kaplan J.A., Grinstaff M.W., Parquette J.R. (2015). Photo-crosslinking of a self-assembled coumarin-dipeptide hydrogel. New J. Chem..

[152] Givens R.S., Rubina M., Wirz J. (2012). Applications of p-hydroxyphenacyl (pHP) and coumarin-4-ylmethyl photoremovable protecting groups. Photochem. Photobiol. Sci..

[153] Ngwuluka N. (2010). Application of in situ polymerization for design and development of oral drug delivery systems. AAPS PharmSciTech.

[154] Williams C.G., Malik A.N., Kim T.K., Manson P.N., Elisseeff J.H. (2005). Variable cytocompatibility of six cell lines with photoinitiators used for polymerizing hydrogels and cell encapsulation. Biomaterials.

[155] Bryant S.J., Nuttelman C.R., Anseth K.S. (2000). Cytocompatibility of UV and visible light photoinitiating systems on cultured NIH/3T3 fibroblasts in vitro. J. Biomater. Sci. Polym. Ed..

[156] Pereira R.F., Bártolo P.J. (2015). 3D Photo-Fabrication for Tissue Engineering and Drug Delivery. Engineering.

[157] Hillel A.T., Unterman S., Nahas Z., Reid B., Coburn J.M., Axelman J., Chae J.J., Guo Q., Trow R., Thomas A. (2011). Photoactivated Composite Biomaterial for Soft Tissue Restoration in Rodents and in Humans. Sci. Transl. Med..

[158] Zhang H., Patel A., Gaharwar A.K., Mihaila S.M., Iviglia G., Mukundan S., Bae H., Yang H., Khademhosseini A. (2013). Hyperbranched polyester hydrogels with controlled drug release and cell adhesion properties. Biomacromolecules.

[159] Milo R., Phillips R. (2015). Cell Biology by the Numbers.

[160] Ortega A.L., Mena S., Estrela J.M. (2011). Glutathione in cancer cell death. Cancers.

[161] Gamcsik M.P., Kasibhatla M.S., Teeter S.D., Colvin O.M. (2012). Glutathione levels in human tumors. Biomarkers.

[162] Su Y., Hu Y., Du Y., Huang X., He J., You J., Yuan H., Hu F. (2015). Redox-responsive polymer-drug conjugates based on doxorubicin and chitosan oligosaccharide- g -stearic acid for cancer therapy. Mol. Pharm..

[163] Nehate C., Nayal A., Koul V. (2018). Redox Responsive Polymersomes for Enhanced Doxorubicin Delivery. ACS Biomater. Sci. Eng..

[164] Thambi T., Park J.H., Lee D.S. (2016). Stimuli-responsive polymersomes for cancer therapy. Biomater. Sci..

[165] Zhi X., Liu P., Li Y., Li P., Yuan J., Lin J. (2018). One-step fabricated keratin nanoparticles as pH and redox-responsive drug nanocarriers. J. Biomater. Sci. Polym. Ed..

[166] Li Q., Yang S., Zhu L., Kang H., Qu X., Liu R., Huang Y. (2015). Dual-stimuli sensitive keratin graft PHPMA as physiological trigger responsive drug carriers. Polym. Chem..

[167] Senapati S., Mahanta A.K., Kumar S., Maiti P. (2018). Controlled drug delivery vehicles for cancer treatment and their performance. Signal Transduct. Target. Ther..

[168] Guo X., Cheng Y., Zhao X., Luo Y., Chen J., Yuan W.-E. (2018). Advances in redox-responsive drug delivery systems of tumor microenvironment. J. Nanobiotechnology.

[169] Pietschnig R. (2016). Polymers with pendant ferrocenes. Chem. Soc. Rev..

[170] Wu J., Wang L., Yu H., Zain-ul-Abdin, Khan R.U., Haroon M. (2017). Ferrocene-based redox-responsive polymer gels: Synthesis, structures and applications. J. Organomet. Chem..

[171] Chen J., Huang Y., Ma X., Lei Y. (2018). Functional self-healing materials and their potential applications in biomedical engineering. Adv Compos Hybrid Mater.

[172] Taylor D.L., in het Panhuis M. (2016). Self-Healing Hydrogels. Adv. Mater..

[173] Nakahata M., Takashima Y., Yamaguchi H., Harada A. (2011). Redox-responsive self-healing materials formed from host-guest polymers. Nat. Commun..

[174] Fang Y., Wang C.F., Zhang Z.H., Shao H., Chen S. (2013). Robust self-healing hydrogels assisted by cross-linked nanofiber networks. Sci. Rep..

[175] Wajs E., Nielsen T.T., Larsen K.L., Fragoso A. (2016). Preparation of stimuli-responsive nano-sized capsules based on cyclodextrin polymers with redox or light switching properties. Nano Res..

[176] Greene A.F., Danielson M.K., Delawder A.O., Liles K.P., Li X., Natraj A., Wellen A., Barnes J.C. (2017). Redox-Responsive Artificial Molecular Muscles: Reversible Radical-Based Self-Assembly for Actuating Hydrogels. Chem. Mater..

[177] Tamesue S., Noguchi S., Kimura Y., Endo T. (2018). Reversing Redox Responsiveness of Hydrogels due to Supramolecular Interactions by Utilizing Double-Network Structures. ACS Appl. Mater. Interfaces.

[178] Riba-Moliner M., Gómez-Rodríguez A., Amabilino D.B., Puigmartí-Luis J., González-Campo A. (2016). Functional supramolecular tetrathiafulvalene-based films with mixed valences states. Polymer (Guildf)..

[179] Schröder H.V., Schalley C.A. (2018). Tetrathiafulvalene – a redox-switchable building block to control motion in mechanically interlocked molecules. Beilstein J. Org. Chem..

[180] Zhang X., Zeng Y., Yu T., Chen J., Yang G., Li Y. (2014). Tetrathiafulvalene terminal-decorated PAMAM dendrimers for triggered release synergistically stimulated by redox and CB[7]. Langmuir.

[181] Bigot J., Charleux B., Cooke G., Fournier D., Woisel P. (2010). Poly (N-isopropylacrylamide): A New Class of Amphiphilic Polymer for the Creation of Multistimuli Responsive Micelles. J. Am. Chem. Soc..

[182] Ning C., Zhou Z., Tan G., Zhu Y., Mao C. (2018). Electroactive polymers for tissue regeneration: Developments and perspectives. Prog. Polym. Sci..

[183] Clancy K.F.A., Hardy J.G. (2017). Gene Delivery with Organic Electronic Biomaterials. Curr. Pharm. Des..

[184] Svirskis D., Travas-Sejdic J., Rodgers A., Garg S. (2010). Electrochemically controlled drug delivery based on intrinsically conducting polymers. J. Control. Release.

[185] Zhao Y.H., Niu C.M., Shi J.Q., Wang Y.Y., Yang Y.M., Wang H.B. (2018). Novel conductive polypyrrole/silk fibroin scaffold for neural tissue repair. Neural Regen. Res..

[186] Guex A.G., Puetzer J.L., Armgarth A., Littmann E., Stavrinidou E., Giannelis E.P., Malliaras G.G., Stevens M.M. (2017). Highly porous scaffolds of PEDOT:PSS for bone tissue engineering. Acta Biomater..

[187] Gelmi A., Ljunggren M.K., Rafat M., Jager E.W.H. (2014). Influence of conductive polymer doping on the viability of cardiac progenitor cells. J. Mater. Chem. B.

[188] Baumgartner J., Jönsson J.I., Jager E.W.H. (2018). Switchable presentation of cytokines on electroactive polypyrrole surfaces for hematopoietic stem and progenitor cells. J. Mater. Chem. B.

[189] Balint R., Cassidy N.J., Cartmell S.H. (2014). Conductive polymers: Towards a smart biomaterial for tissue engineering. Acta Biomater..

[190] Fortunato M.G., De Maria C., Eglin D., Serra T., Vozzi G. (2018). An ink-jet printed electrical stimulation platform for muscle tissue regeneration. Bioprinting.

[191] Pires F., Ferreira Q., Rodrigues C.A.V., Morgado J., Ferreira F.C. (2015). Neural stem cell differentiation by electrical stimulation using a cross-linked PEDOT substrate: Expanding the use of biocompatible conjugated conductive polymers for neural tissue engineering. Biochim. Biophys. Acta Gen. Subj..

[192] Hoop M., Chen X.Z., Ferrari A., Mushtaq F., Ghazaryan G., Tervoort T., Poulikakos D., Nelson B., Pané S. (2017). Ultrasound-mediated piezoelectric differentiation of neuron-like PC12 cells on PVDF membranes. Sci. Rep..

[193] Vannozzi L., Ricotti L., Filippeschi C., Sartini S., Coviello V., Piazza V., Pingue P., La Motta C., Dario P., Menciassi A. (2015). Nanostructured ultra-thin patches for ultrasound-modulated delivery of anti-restenotic drug. Int. J. Nanomedicine.

[194] Kim O., Shin T.J., Park M.J. (2013). Fast low-voltage electroactive actuators using nanostructured polymer electrolytes. Nat. Commun..

[195] Shin S.R., Jung S.M., Zalabany M., Kim K., Zorlutuna P., Kim S.B., Nikkhah M., Khabiry M., Azize M., Kong J. (2013). Carbon-nanotube-embedded hydrogel sheets for engineering cardiac constructs and bioactuators. ACS Nano.

[196] Chen Z., Wu C., Zhang Z., Wu W., Wang X., Yu Z. (2018). Synthesis, functionalization, and nanomedical applications of functional magnetic nanoparticles. Chinese Chem. Lett..

[197] Tuttolomondo M.V., Villanueva M.E., Alvarez G.S., Desimone M.F., Díaz L.E. (2013). Preparation of submicrometer monodispersed magnetic silica particles using a novel water in oil microemulsion: properties and application for enzyme immobilization. Biotechnol. Lett..

[198] Zhao Y., Fan T., Chen J., Su J., Zhi X., Pan P., Zou L., Zhang Q. (2019). Magnetic bioinspired micro/nanostructured composite scaffold for bone regeneration. Colloids Surfaces B Biointerfaces.

[199] Ridi F., Bonini M., Baglioni P. (2014). Magneto-responsive nanocomposites: Preparation and integration of magnetic nanoparticles into films, capsules, and gels. Adv. Colloid Interface Sci..

[200] Abu-Dief A.M., Abdel-Fatah S.M. (2018). Development and functionalization of magnetic nanoparticles as powerful and green catalysts for organic synthesis. Beni-Suef Univ. J. Basic Appl. Sci..

[201] Kayode B., Abdul A. (2016). Journal of Magnetism and Magnetic Materials Recent advances in synthesis and surface modi fi cation of superparamagnetic iron oxide nanoparticles with silica. J. Magn. Magn. Mater..

[202] Silva Diorato Teixeira de Mendonça E., Britto de Faria A.C., Loureiro Dias S.C., Aragón F.F.H., Mantilla J.C., Coaquira J.A.H., Dias J.A. (2018). Effects of silica coating on the magnetic properties of magnetite nanoparticles. Surfaces and Interfaces.

[203] Zhang Y., Zhen B., Li H., Feng Y. (2018). Preparation of water-soluble magnetic nanoparticles with controllable silica coating. Chinese J. Chem. Eng..

[204] Bui T.Q., Ngo H.T.M., Tran H.T. (2018). Surface-protective assistance of ultrasound in synthesis of superparamagnetic magnetite nanoparticles and in preparation of mono-core magnetite-silica nanocomposites. J. Sci. Adv. Mater. Devices.

[205] Hou Y., Sellmyer D.J. (2017). Magnetic Nanomaterials: Fundamentals, Synthesis and Applications.

[206] Basith M.A., Quader A., Rahman M.A., Ngo D.-T., Mølhave K., Sinha B.L., Ahmmad B., Hirose F. (2014). Simple top-down preparation of magnetic Bi0.9Gd0.1Fe1−xTixO3 nanoparticles by ultrasonication of multiferroic bulk material. Nanoscale.

[207] Laurent S., Forge D., Port M., Roch A., Robic C., Elst L.V., Muller R.N. (2008). Magnetic Iron Oxide Nanoparticles: Synthesis, Stabilization, Vectorization, Physicochemical Characterizations, and Biological Applications. Chem. Rev..

[208] Gul S., Khan S.B., Rehman I.U., Khan M.A., Khan M.I. (2019). A Comprehensive Review of Magnetic Nanomaterials Modern Day Theranostics. Front. Mater..

[209] Frey N.A., Peng S., Cheng K., Sun S. (2009). Magnetic nanoparticles: synthesis, functionalization, and applications in bioimaging and magnetic energy storage. Chem. Soc. Rev..

[210] Schmid G., Fenske D. (2010). Metal clusters and nanoparticles. Philos. Trans. R. Soc. A Math. Phys. Eng. Sci..

[211] Neoh K.G., Kang E.T. (2012). Surface modification of magnetic nanoparticles for stem cell labeling. Soft Matter.

[212] Schladt T.D., Schneider K., Schild H., Tremel W. (2011). Synthesis and bio-functionalization of magnetic nanoparticles for medical diagnosis and treatment. Dalt. Trans..

[213] Thong-On B., Rutnakornpituk B., Wichai U., Rutnakornpituk M. (2012). Magnetite nanoparticle coated with amphiphilic bilayer surfactant of polysiloxane and poly(poly(ethylene glycol) methacrylate). J. Nanoparticle Res..

[214] Wang X., Tilley R.D., Watkins J.J. (2014). Simple Ligand Exchange Reactions Enabling Excellent Dispersibility and Stability of Magnetic Nanoparticles in Polar Organic, Aromatic, and Protic Solvents. Langmuir.

[215] Lassalle V., Agotegaray M. (2017). Silica-Coated Magnetic Nanoparticles: An Insight into Targeted Drug Delivery and Toxicology.

[216] Gao J., Gu H., Xu B. (2009). Multifunctional Magnetic Nanoparticles: Design, Synthesis, and Biomedical Applications. Acc. Chem. Res..

[217] Singh D., Mcmillan J.E.M., Kabanov A.V., Sokolsky-Papkov M., Gendelman H.E. (2014). Bench-to-bedside translation of magnetic nanoparticles. Nanomedicine.

[218] Xia Y., Sun J., Zhao L., Zhang F., Liang X.J., Guo Y., Weir M.D., Reynolds M.A., Gu N., Xu H.H.K. (2018). Magnetic field and nano-scaffolds with stem cells to enhance bone regeneration. Biomaterials.

[219] Lu J.-W., Yang F., Ke Q.-F., Xie X.-T., Guo Y.-P. (2018). Magnetic nanoparticles modified-porous scaffolds for bone regeneration and photothermal therapy against tumors. Nanomedicine Nanotechnology, Biol. Med..

[220] Rotherham M., Henstock J.R., Qutachi O., El Haj A.J. (2018). Remote regulation of magnetic particle targeted Wnt signaling for bone tissue engineering. Nanomedicine Nanotechnology, Biol. Med..

[221] Ito A., Kamihira M. (2011). Tissue engineering using magnetite nanoparticles. Prog. Mol. Biol. Transl. Sci..

[222] Rao K.M., Kumar A., Han S.S. (2018). Polysaccharide-based magnetically responsive polyelectrolyte hydrogels for tissue engineering applications. J. Mater. Sci. Technol..

[223] Municoy S., Ibañez I.L., Durán H., Bellino M.G. (2014). A catalase-magnetic switch for cell proliferation. RSC Adv..

[224] Mørup S., Hansen M.F., Frandsen C. (2018). Magnetic Nanoparticles. Reference Module in Materials Science and Materials Engineering.

[225] Zavisova V., Koneracka M., Gabelova A., Svitkova B., Ursinyova M., Kubovcikova M., Antal I., Khmara I., Jurikova A., Molcan M. (2019). Effect of magnetic nanoparticles coating on cell proliferation and uptake. J. Magn. Magn. Mater..

[226] Alipour A., Soran-Erdem Z., Utkur M., Sharma V.K., Algin O., Saritas E.U., Demir H.V. (2018). A new class of cubic SPIONs as a dual-mode T1 and T2 contrast agent for MRI. Magn. Reson. Imaging.

[227] Yang H.M., Park C.W., Park S., Kim J.D. (2018). Cross-linked magnetic nanoparticles with a biocompatible amide bond for cancer-targeted dual optical/magnetic resonance imaging. Colloids Surfaces B Biointerfaces.

[228] Rodrigues G.R., López-Abarrategui C., de la Serna Gómez I., Dias S.C., Otero-González A.J., Franco O.L. (2019). Antimicrobial magnetic nanoparticles based-therapies for controlling infectious diseases. Int. J. Pharm..

